# Oxidative Stress as the Main Target in Diabetic Retinopathy Pathophysiology

**DOI:** 10.1155/2019/8562408

**Published:** 2019-08-14

**Authors:** Olvera-Montaño Cecilia, Castellanos-González José Alberto, Navarro-Partida José, Cardona-Muñoz Ernesto Germán, López-Contreras Ana Karen, Roman-Pintos Luis Miguel, Robles-Rivera Ricardo Raúl, Rodríguez-Carrizalez Adolfo Daniel

**Affiliations:** ^1^Institute of Clinical and Experimental Therapeutics, Department of Physiology, Health Sciences University Center, University of Guadalajara, Mexico; ^2^Department of Ophthalmology, Specialties Hospital of the National Occidental Medical Center, Mexican Institute of Social Security, Mexico; ^3^Tecnológico de Monterrey Institute, School of Medicine and Health Sciences, Campus Guadalajara, Mexico; ^4^Department of Physiology, University Center of Tonalá, University of Guadalajara, Mexico

## Abstract

Diabetic retinopathy (DR) is one of the most common complications of diabetes mellitus (DM) causing vision impairment even at young ages. There are numerous mechanisms involved in its development such as inflammation and cellular degeneration leading to endothelial and neural damage. These mechanisms are interlinked thus worsening the diabetic retinopathy outcome. In this review, we propose oxidative stress as the focus point of this complication onset.

## 1. Introduction

Diabetes mellitus (DM) is expected to affect around 550 million people all over the world according to global estimates of the prevalence of diabetes [1]. DM is characterized by constant hyperglycemia that damages various organs and manifests in macrovascular complications like premature atherosclerosis resulting in strokes, peripheral vascular disease, and myocardial infarctions and microvascular complications such as nephropathy, neuropathy, and retinopathy [2].

Diabetic retinopathy (DR) is the number one cause of blindness in people between 27 and 75 years of age. Prevalence of DR is around 25% and 90% at 5 and 20 years, respectively, from diagnosis; it is calculated that 191 million people will be diagnosed with this microvascular complication by the year 2030 [3]. It consists of progressive retinal structure and function loss due to vessel damage such producing blood-retina barrier rupture and promoting new vessel formation in the presence of chronic hyperglycemia [4].

The first clinical signs of DR are microaneurysms in the retina found in the mild version of the disease. In moderate diabetic retinopathy, exudates, hemorrhages, and minimum intraretinal microvascular abnormalities are present up to being prominent in severe stages among with more than 20 hemorrhages and venous rosaries in at least 2 quadrants. Neovascularization is the main clinical change in proliferative diabetic retinopathy (PDR) [5].

Through the last three decades, extensive scientific reports have shown ROS to play an important role in DM complications such as diabetic neuropathy, nephropathy, and retinopathy due to alterations on the biomechanisms involved in the instauration and progression of microvascular complications [6]. These three microvascular complications share high glucose levels as a starting point; nonetheless, according to Barret et al., such condition is necessary, but may not be enough to initiate the damage present in the peripheral nervous system (neuropathy), kidneys (nephropathy), and retinas (retinopathy) of diabetic patients [7, 8]. In addition, the activation of various pathways involving proinflammatory factors and reactive oxygen species overproduction has been linked to vascular injury in the structures previously mentioned [9–11]. With this in mind, multiple molecules and nutraceuticals have been studied in recent years by their antioxidant effects due to their apparent benefits over diabetes and its complications [12–15].

As will be seen in this document, hyperglycemic states favor the activation of alternative pathways leading to reactive oxygen species (ROS) formation and augmented concentrations locally and in the rest of the body even at the point of surpassing the antioxidant capacity, a state known as oxidative stress affecting retinal integrity.

## 2. Pathophysiology of Diabetic Retinopathy

**Figure 1:**
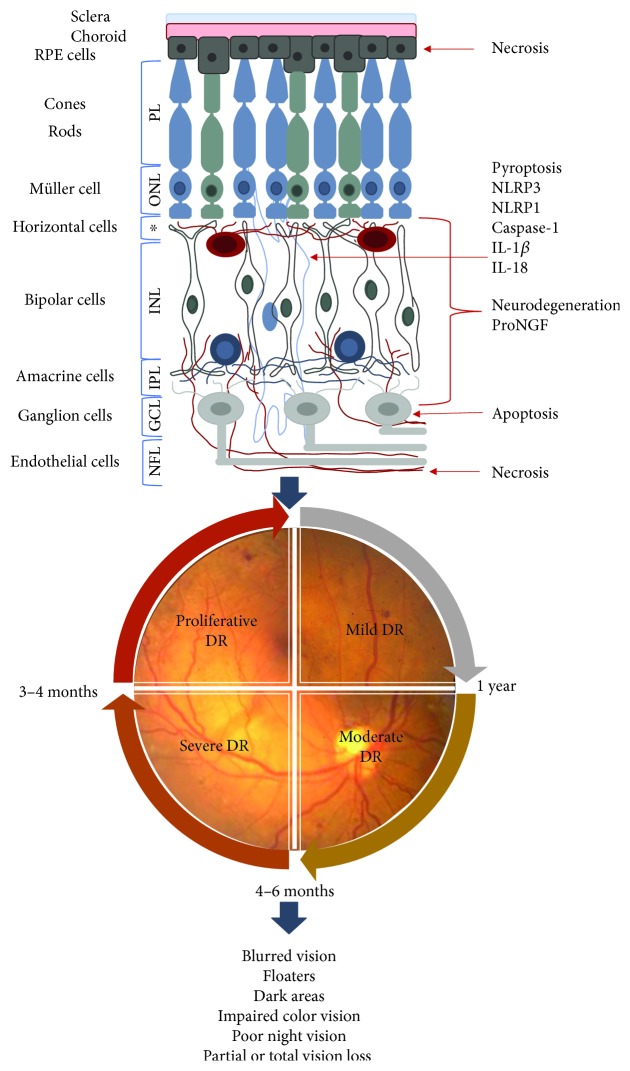
Damage at each retinal layer. A series of events occur in early DR development. Neurodegeneration of horizontal, bipolar, amacrine, and ganglion cells. These damages may be determined by proNGF concentrations as NLRP3 and NLRP1 are related to eye degenerative diseases. NFL: nerve fiber layer; GCL: ganglion cell layer; IPL: inner plexiform layer; INL: inner nuclear layer; ^∗^OPL: outer plexiform layer; ONL: outer nuclear layer; PL: photoreceptor layer.

The retina is a high energy-demanding organ, which makes it susceptible to high levels of free radicals or ROS. Multiple factors are implicated in DR pathophysiology. Along with hyperglycemia that promotes changes in vascular and neuronal structures through ischemic or hyperosmotic damage, it also leads to oxidative stress (OS). Oxidative stress produces inflammation, mitochondrial dysfunction, and cell death, via pyroptosis, apoptosis or autophagia, and neurodegeneration that in conjunction leads to neural, vascular, and retinal tissue damage. In recent years, it has been found that such damages are present in a sequential order, in which neurodegeneration takes place before microvascular dysfunction, then clinical characteristics may be found, and finally symptoms appear. However, one could believe that these steps occur in a timely manner and that each biomechanism happens only in one direction; study findings show that different biomechanisms are active at the same time and have an influence between them. As seen in Figure 1, the retina consists different types of cells that form identifiable layers, from the endothelial layer in the inner side of the eye through the retinal pigmented cell layer in the outer side close to the choroidal surface. At each layer, various biomechanisms such as inflammation, pyroptosis, and neurodegeneration could appear simultaneously and have an intricate relationship with high levels of reactive oxygen species and oxidative stress.

### 2.1. Hyperglycemia in Diabetic Retinopathy

**Figure 2:**
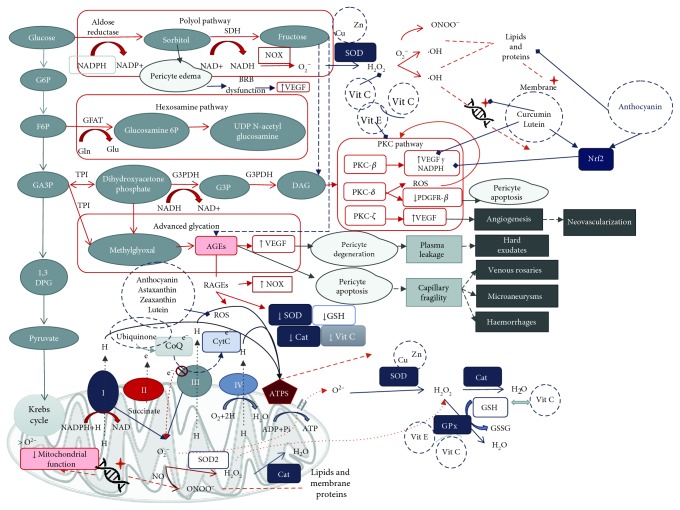
Glucose metabolic pathways in the hyperglycemic milieu, oxidative stress in diabetic retinopathy, and antioxidant targets. In hyperglycemic states, different pathways were activated producing ROS which enhance inflammatory, apoptotic, and degeneration pathways, ultimately leading to the appearance of diabetic retinopathy clinical characteristics. Some antioxidant substances are able to interact with ROS (xanthophylls, vitamins C and E, and anthocyanin); others function as cofactors to enhance antioxidant enzymes (Cu, Zn, and vitamins E and C), and others are capable of inhibiting the expression of proinflammatory and prodegeneration factors (curcumin and lutein). Finally, all of them interfere in diabetic retinopathy development.

Through the glycolytic pathway, glucose suffers various biotransformations up to pyruvate that enters the Krebs cycle in the mitochondria to follow the respiratory chain in order to synthesize adenosine triphosphate (ATP). It is known that high concentrations of serum glucose can cause damage to cell structure and function. In the retina, pericytes are key cells in normal retinal function. As shown in Figure 2, these cells suffer from edema due to intracellular accumulation of sorbitol, which is formed by aldose reductase in the presence of high blood sugar through the polyol pathway, leading to a blood-retinal barrier (BRB) dysfunction [16, 17]. Edema causes vessels to swallow impeding adequate perfusion especially in the inner retina where blood supply is sparse compared to the outer retina [18]. Ischemia upregulates the expression of vascular endothelial growth factor (VEGF), known to play a role in angiogenesis, increased permeability, and activation of proinflammatory proteins [19]. All of them are important mechanisms involved in the development of diabetic retinopathy [18, 19]. On the other hand, the presence of glucose forms glyceraldehyde-3 phosphate (DHAP) through the glycolysis pathway; these two phosphates are very reactive to the nonenzymatic formation of methylglyoxal (MG) [20]. Such dicarbonyl (methylglyoxal) has been implicated in the activation of the hexosamine pathway, loss of pericytes, and decreased function of bipolar cells in the retina even in the absence of hyperglycemia [21]. The hexosamine pathway transforms fructose 6-phosphate into UDP-N-acetyl glucosamine (UDP-GlcNAc). When this very last molecule exceeds its normal concentrations, it promotes protein modifications by O-glycosyl-N-acetylation (O-GlcNAc) inducing an exacerbated activity; one of those proteins is nuclear factor-*κ*B (NF-*κ*B), a factor known to be implicated in DR worsening [22–24].

Methylglyoxal activates the advanced glycation pathway, AGE formation, and receptor activation (RAGE). AGEs can promote VEGF activation which alters tight junctions between retinal pigmented endothelial (RPE) cells. Such alterations lead to increased vascular permeability and leakage of blood components into the retina [25]. VEGF also mediates angiogenesis, so when chronic hyperglycemia persists, this factor deviates from physiological functions onto the formation of pathologic new vessels as happens in proliferative diabetic retinopathy among other cytokines, proinflammatory, proangiogenic, and prooxidative factors [26].

Hyperglycemia augments thioredoxin-interactin protein (TXNIP) levels, an inflammation mediator in Müller glia. TXNIP upregulation activates cellular defense mechanisms including autophagy, hypoxic-like HIF-1*α* induction and inflammasome formation [27].

According to many studies, the principal cause of DR is the lack of or poor glycemic control, but hypertension and dyslipidemia management has been proven to be beneficial in reducing progression and incidence of this complication [28, 29].

### 2.2. Reactive Oxygen Species in Diabetic Retinopathy

ROS are free radicals, oxidant molecules that contain one extra electron conferring them great instability and reactivity. By trying to regain stability, they obtain electrons from other molecules in the vicinity, therefore creating an oxidative chain [30].

As presented in Figure 2, ROS are formed in a physiological manner through the electron transport chain in the mitochondria derived from oxygen; some of the most common ROS are superoxide anion (O^•^), hydrogen peroxide (H_2_O_2_), and hydroxyl radical (OH^−^) [31]. By antioxidant enzymatic defenses, such as catalase, glutathione peroxidase, superoxide dismutase, hemoxygenase 1, peroxiredoxins, and glutaredoxins, and nonenzymatic antioxidants, the body is capable of maintaining a redox balance. When the production of ROS is higher than the antioxidant defenses, OS occurs and, at that point, cellular and mitochondrial function get affected [17, 32].

OS has been considered one of the most important factors in the development of DR and chronic hyperglycemia and also plays a role in the formation of ROS due to the activation of the secondary pathways like the polyol and the protein kinase C (PKC) and overactivity of the hexosamine pathways [32, 33].

Glucose metabolism is known to involve redox reactions as the main purpose in energy production by extraction, storage, and transport of electrons. When glycemic conditions are normal, glucose undergoes transformation through the glycolysis pathway to produce ATP by the Krebs cycle in the mitochondria, where electrons are stored in NADH and FADH2. Then, in the respiratory chain, they donate the electrons to the complex I or complex II. In complex IV, oxygen is used again to receive electrons from cytochrome c [34]. Nonetheless, the polyol pathway is increased during diabetes; it consumes 30% of the systemic glucose. It consists of the production of sorbitol by two main reactions dependent of aldose reductase (AR) and sorbitol dehydrogenase (SDH) by the consumption of NADPH [34]. As mentioned before, sorbitol leads to osmotic stress and damage in the capillaries; also, in the reaction of converting sorbitol to fructose by SDH, reduced Nicotinamide Adenine Dinucleotide (NADH) is formed. As we can see in Figure 2, NADH now serves as substrate of Nox family enzymes to produce superoxide [35], contributing to redox imbalance and oxidative stress. Another way that NADH may contribute to the redox imbalance is by reductive stress creating pseudohypoxia and overwhelming mitochondria complex I function [36]. Complex I is not able to oxidize all NADH available, though by trying, it pumps more electrons to partially reduce oxygen leading to superoxide formation instead of adequate usage of oxygen and electrons [37]. In this case, NADH concentrations would still be higher than NAD+ which is needed to transport electrons to oxygen; this alteration in the appropriate consumption of oxygen is known as pseudohypoxia [38].

Fructose upregulates the formation of AGE [34, 39]. Endogenous fructose from the polyol pathway (Figure 2) suffers a rearrangement in carbon 2 by a reaction called Heyns reaction. Afterwards, the products undergo processes of rearrangement, dehydration, and condensation to form AGEs. By the Maillard reaction and Amadori rearrangement, glucose ends up forming AGEs yet the fructose-specific AGEs have not been yet described [40].

When the polyol pathway is activated during diabetes, OS is increased, then the increase in the activity of the polyol pathway is postulated to deplete NADPH by competing with glutathione reductase, and the availability of NADPH may be reduced and less available to regenerate intracellular antioxidants [41]. Accordingly, NADPH and ATP are decreased in lenses of diabetic rats with higher concentrations of sorbitol and fructose than healthy rats, supporting the findings on the activation of the polyol pathway in sustained hyperglycemic states [42, 43].

Through the hexosamine pathway, glutamine:fructose-6-phosphate amidotransferase (GFAT) oxidizes glutathione as a cofactor in order to transform F6P into glucosamine-6-phosphate; GFAT activity is significantly higher in diabetic subjects inducing to a lower pool of such endogenous antioxidant (glutathione) [44].

Diacylglycerol (DAG) is formed from 6-phosphate dihydroxyacetone phosphate, the second metabolite from fructose 6-phosphate (from the polyol pathway or glycolysis). DAG, in turn, activates the PKC pathway. PKCs are calcium and DAG-dependent kinases; the activation of these molecules has been associated to increased vascular permeability and abnormal angiogenesis in hyperglycemic and hypoxic conditions [45, 46].

PKC*β* and PKC*ς* are involved in the VEGF-dependent retinal barrier changes [47]. PKC*β* also increases the activity of NADPH oxidase that produces superoxide [48, 49]. On the other hand, activation and translocation of PKC*δ* have proven to promote proliferation in the retinal tissue even in the absence of hypoxia [46]. In cell cultures, PKC*δ* activation by phosphorylation is able to inactivate complex IV of the mitochondria, thus augmenting ROS production [50].

At high glucose levels, glyceraldehyde-3-phosphate transforms to methylglyoxal, a precursor of AGE formation which is implicated in pericyte apoptosis and VEGF elevation. The activation of receptors for AGEs (RAGEs) leads to Nox augmentation, increase of ROS production, and decrease in SOD, catalase, glutathione, and vitamin C antioxidant activities [51] (see Figure 2).

Next, we discuss the following biomechanisms implicated in DR that have been described to be upregulated or closely related to oxidative stress, from inflammation to neurodegeneration.

### 2.3. ROS, Inflammation, and Pyroptosis in Diabetic Retinopathy

It has been proven that diabetes is an inflammatory state since hyperglycemia leads to cell malfunction and elevation of several cytokines and inflammatory mediators. Reactive oxygen species such as H_2_O_2_ and superoxide anion promote NF-*κ*B production which in turn mediates VEGF expression; at the same time, it is activated by VEGF and translocated to the nucleus to promote the expression of proinflammatory mediators such as ICAM-1, vascular cell adhesion molecule-1 (VCAM-1), monocyte chemotactic protein-1 (MCP-1), and cyclooxygenase-2 (COX-2) [19, 52]. It is known that COX-2 increases prostaglandin synthesis; prostaglandins stabilize hypoxia-induced factor-1 (HIF-1) which favors VEGF expression and NF-*κ*B activation for COX-2 expression. This way an inflammatory mediator loop is formed [53–56]. ICAM-1, VCAM-1, and VEGF are implicated in BRB disruption that causes microaneurysms and leakage in the retina [57].

As inflammatory factors are activated, an inflammasome is formed recruiting the adaptor apoptosis speck-like protein containing a CARD (ASC); this cleaves caspase-1 activating IL-1*β* and IL-18 and leading to cell death, and this particular death process that includes damage and rupture of the cell membrane is known as pyroptosis [58].

Pyroptosis is a type of caspase-1-dependent death cleaved by inflammatory molecular platforms called inflammasomes, also called pyroptosomes [59]. Such platforms contain oligomers of ASC adaptor proteins with a sensor of danger-associated molecular patterns (DAMPs) or pathogen-associated molecular patterns (PAMPs) [59] and are assembled by a variety of toll-like receptors (TLRs), either one of the six nod-like receptors (NLRs) or IFN*γ*-inducible protein absent in melanoma 2 (AIM2) or retinoic acid-inducible gene-I- (RIG-I-) like helicase. According to recent studies, inflammasomes NLRP3 (NOD-like receptor pyrin domain-containing 3) and NLRP1 (NOD-like receptor pyrin domain-containing 1) are associated to retinal diseases [58, 60].

**Figure 3:**
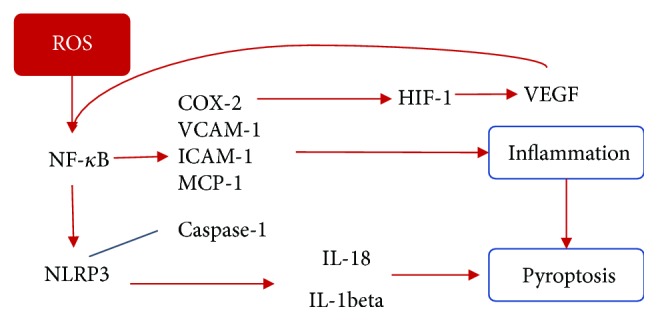
The ROS role in inflammation and pyroptosis. ROS augments NF-*κ*B production which promotes proinflammatory mediators favoring the expression of VEGF. VEGF translocates NF-*κ*B into the nucleus, and NF-*κ*B activate NLRP3 with caspase cleavage leading to cytokine release. NLRP3 inflammasome has been associated to diabetic retinopathy by Müller pyroptosis by the caspase-1/IL-1beta pathway. NF-*κ*B: nuclear factor kappa B; COX-2: cyclooxygenase-2; VEGF: vascular endothelial growth factor.

It has been explained how ROS have a very important role in the DR development and progression, as synthesized in Figure 3; they also promote the assembly of inflammasomes or pyroptosomes leading to pyroptosis [58] (see Figure 3).

### 2.4. ROS and Autophagy in Diabetic Retinopathy

Diabetes is related to different forms of cell death (apoptosis, pyroptosis, and autophagic death) affecting retinal cells like pericytes, ganglion, and Müller cells [61]. In retinal cells, apoptosis may be triggered by the excess of ROS that upregulate matrix metalloproteinases 9 and 2 (MMP-9 and MMP-2) which impair mitochondrial membrane potential leading to apoptosis via mitochondrial pathway [62].

Autophagy is the process of degradation and recycling of proteins and organelles. Autophagy's main function is to regulate processes such as maintenance of organelle integrity, control of protein quality, and regulation of stress and immune responses [63]. Two forms of autophagy may be cited: (a) nonselective autophagy, triggered by nutrient deficiency in order to acquire metabolic components, and (b) cargo-specific autophagy, employed to remove impaired or nonfunctional organelles like ribophagy (ribosome elimination), pexophagy (peroxisome elimination), and mitophagy (mitochondrial removal). There are three types of autophagy in mammalian cells: (1) macroautophagy, (2) chaperone-mediated autophagy, and (3) microautophagy [61].

Macroautophagy is basically done in four steps: (1) ubiquitination labeling of the molecules or structures to be recycled, (2) autophagosome formation, (3) fusion to lysosomes (autophagolysosomes) that provide hydrolytic enzymes, and (4) release of products. It consists of the sequestration of the cargo (organelles and macromolecules) into the lysosome [64, 65].

Chaperone-mediated autophagy transports the cargo (protein complexes or unfolded proteins) across the lysosomal membrane while microautophagy uptakes the cargo (protein remains or small molecules) into the lysosome via an invagination, without a phagosome formation [65].

**Figure 4:**
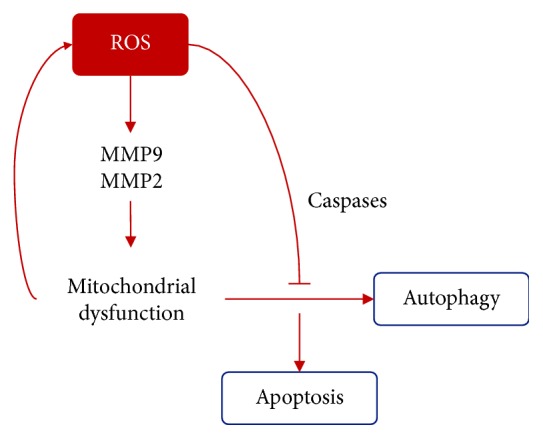
The ROS role in autophagy. ROS upregulate MMP9 and MMP2 that leads to mitochondrial membrane potential impairment. When a mithochondrion malfunctions, autophagy (mitophagy) is activated, though in high stress conditions, caspases inactivate mitophagy and activate apoptosis pathways.

Macroautophagy is activated under normal conditions to maintain cellular homeostasis though it is also induced by stress conditions whether it is starvation or OS to protect the cell [66]. In diabetes, an overload to the mitochondria leads to mitochondria dysfunction (MD) which is the loss of efficiency in the electron transport chain; it promotes ROS production creating a vicious cycle in which ROS damage mitochondrial structures and machinery; when the cell detects this malfunctioning, it induces mitophagy to survive [11]. At high oxidative stress levels, caspases inactivate autophagy and activate apoptosis [67] (see Figure 4). Moreover, it has been shown that autophagy deficiency in beta cells creates a reduced insulin production but chronic activation of autophagy leads to autophagic cell death [63, 68].

As mentioned above, ROS production, hyperglycemic states, and ischemia are implicated in the upregulation of VEGF. This growth factor activates mammalian target of rapamycin (mTOR) which in physiological conditions prevents autophagy promoting RPE cell dedifferentiation and photoreceptor preservation, though in energy deficiency intracellular conditions, whether by lack of ATP (mitochondrial dysfunction) or lack of glucose (vascular disruption), other growth factors such as insulin-like factor induce autophagy via modulation of mTOR/AMPK (AMP-activated protein kinase) by the activation of caspase-3, reduction of glutathione, and photoreceptor cell death [61].

VEGF, ICAM, and nitric oxide have been associated with retinal photoreceptor disruption and severity of diabetic retinopathy. Photoreceptor cells release factors that control neuronal survival and angiogenesis, such as the pigment epithelium-derived factor (PEDF), which promotes the survival of photoreceptors and has an antiangiogenic action [69].

The unbalanced expression of VEGF seems to be implicated in important human pathologies, such as choroidal neovascularization (VNC) in diabetic retinopathy [69]. Vascular endothelial growth factor (VEGF) induces the expression of retinal intercellular adhesion molecule 1 (ICAM-1) and initiates the adhesion of retinal leukocytes, which leads to an early rupture of the retinal barrier and generates no capillary perfusion, injury, and death of endothelial cells [70, 71]. DR causes the interruption of the external limiting membrane (ELM) and the junction of the internal segment and the external segment of the photoreceptor, which is related to DR severity and affects visual acuity [70, 72].

ICAM-1 has been implicated in leukostasis development, a prominent DR feature. Its specific inhibition prevents leukocyte adhesion on the diabetic retina and the rupture of the hematoretinal barrier. ICAM-1 is eliminated by the cell and is the key mediator of the effect of VEGF on retinal leukostasis [70, 73].

The neuronal nitric oxide synthase (NOS) may be responsible for the production of NO in photoreceptors and bipolar cells which has significant effects on the blood flow, neutrophil aggregation, and platelet aggregation [74].

Inducible NOS, found in Müller cells and in the retinal pigment epithelium, can participate in normal phagocytosis of the outer segment of the retina, in infectious and ischemic processes, and in the pathogenesis of the diabetic retinopathy. Nitric oxide is involved in maintaining rest in the uveal and retinal circulations, which contributes to the basal tone in the latter [74, 75]. Retinal ischemia occurs because of a primary ocular disease, such as vascular occlusion of the retina or as a consequence of a systemic disease, such as diabetes mellitus. NO significantly affects the blood flow, neutrophil activation, and platelet aggregation [74].

### 2.5. ROS and Neurodegeneration in Diabetic Retinopathy

Let us recall that the retina is formed by various layers; one of which is the neural retina, composed of ganglion, amacrine, horizontal, and bipolar cells as well as light-sensitive photoreceptors. These cells interact with each other to transmit visual signals to the brain [76]. Neural retina cells are altered in their function in patients with diabetes as many studies in the past have shown. According to a longitudinal study performed by Kim et al., patients with diabetic retinopathy who had at least 2-step progression in a 4-year follow-up presented a greater thinning rate of macular ganglion cell-inner plexiform layer [77].

In the last decade, it has been demonstrated that constant high glucose concentrations lead to death of neurons in the retina even before apoptosis of pericytes begins [78–80]. These alterations may result from hypoxia and inflammation [81]. The retina is a highly energy- and oxygen-demanding tissue; hypoxia is a mechanism known to induce neuronal degeneration [82].

**Figure 5:**
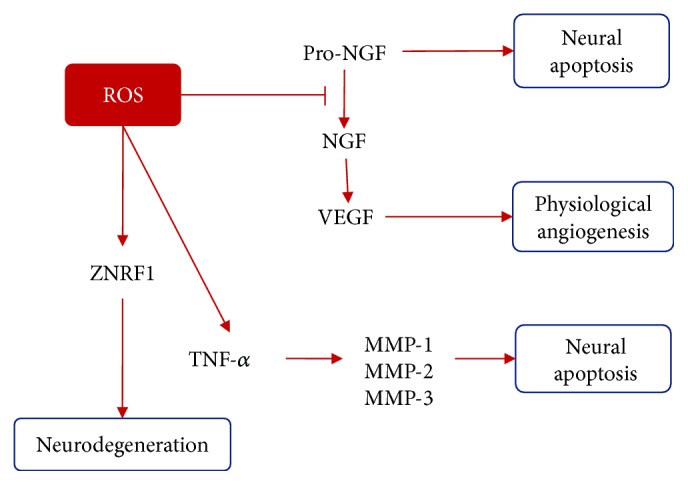
The ROS role in neurodegeneration. In physiological conditions, NGF activates VEGF to promote angiogenesis and protect nerves from hypoxia and ROS inhibits NGF formation from its precursor which leads to neural apoptosis. ROS activate ZNRF1 that provokes neurodegeneration; at the same time, TNF-*α* activates apoptosis via metalloproteinase/caspase pathway. ZNRF1: zinc and ring finger-1; NGF: nerve growth factor; VEGF: vascular endothelial growth factor; MMP: matrix metalloproteinases; TNF-*α*: tumor necrosis factor-*α*.

A number of cytokines and neurotrophic factors related to hypoxia have been described to be implicated in the diabetic retinopathy onset; some of them are also responsible for neurodegeneration [83, 84]. Secretion of IL-1*β*, IL-6, IL-8, MCP-1, TNF-*α*, and VEGF factors known to play an important role in inflammation pathways and pyroptosis may have a role in neurodegeneration as well [85]. As shown in Figure 5, TNF-*α* is also induced by H_2_O_2_ by activating caspases in numerous nerve cells [86]. Oxidative stress has been implicated in axonal degeneration and neuronal apoptosis in traumatic and nontraumatic nerve degeneration, via ZNRF1 activation by oxidative stress [87].

There appear to be some factors that protect amacrine, ganglion, and Müller cells from degradation like brain-derived neurotrophic factor (BDNF), nerve growth factor (NGF), and mesencephalic astrocyte-derived neurotrophic factor (MANF). Müller cells produce NGF increasing the expression of VEGF contributing to angiogenesis in physiological conditions in order to protect neuronal cells from the oxygen-glucose-deprived milieu [88, 89]. Oxidative stress in the retina is capable of preventing NGF activation from its precursor form proNGF which is known to promote apoptosis of neural retina cells [90] (see Figure 5). An imbalance of NGF/proNGF in vitreous correlates to retinal damage [91]. Other factors that may contribute to retinal neuron protection are ciliary neurotrophic factor (CNTF) and fibroblast growth factor (FGF) [92]. Reactive oxygen species lowering has shown to be helpful in protecting neuronal degeneration and favoring the expression of protective factors like compact myelin proteins [93]. Retinal ganglion cell survival is also promoted by PEDF (pigment epithelium-derived factor) via STAT3 (signal transduction and activator of transcription 3) activation secreted by Müller cells [94].

### 2.6. Oxidative Stress-Related Genetics in Diabetic Retinopathy

Studies have shown that DR has a genetic component by observing higher prevalence in certain ethnic groups: Hispanics, Asians, and African Americans.

It is worth noting the complexity of DR as a complication of diabetes and that this is influenced by hereditary factors and the environment [95]. Some DR phenotypes show that changes in the neural retina and the associated microvascular network resulting in abnormal and leaking vessels are a distinctive feature of this pathology [95].

Genetic predisposition of some ethnic groups who suffer from retinopathy is suggested in some studies. It has been found a higher prevalence among Hispanic and African American individuals than in non-Hispanic whites [96, 97]. Knowledge about genetics of this disease will be useful to identify the genome variants that are associated with the higher possibility of complications among individuals with DM; this would allow generating strategies or guidelines for the early identification of diabetic individuals with a high risk of developing DR. In this sense, three main research strategies have been discussed: linkage studies in families, candidate genes, and complete genome association studies (GWAS) [97]. It has been estimated that inheritance is as high as 27% for DR and 52% for PDR [98]. Relatives of patients with DR have a 2-4 times higher risk of developing DR compared to family members of patients without retinopathy [99].

#### 2.6.1. Polymorphisms Linked to DR

It is said that the DR is a complex genetic disease which means it is commonly associated with multiple genetic and environmental factors. These factors are commonly called polymorphisms instead of mutations [100].

Thus, a polymorphism can increase or decrease the risk of suffering from the disease. Some of the advances about DR genetics involve the following genes as part of the DR pathogenesis [101].

*(1) Aldose Reductase (ALR)*. Aldose reductase (ALR) is the first limiting enzyme in the polyol pathway responsible of inducing vascular and hemodynamic pathogenic changes that contribute to DR, as well as the result of sorbitol accumulation, oxidative damage, and protein kinase C activation [102]. ALR is found in high concentrations in Schwann cells and in retinal pericytes where glucose is converted to sorbitol; polymorphisms of ALR have been significantly associated in some populations [100]. Vascular retinal changes, such as the degeneration of retinal pericytes and the development of microaneurysms, can be induced in rats and dogs that have become hyperglycemic with a galactose-rich diet, but galactose is reduced by aldose reductase (AKR1B1) to form galactitol [103]. Consequently, search of pharmacological inhibitors of this enzyme for the treatment of DR is taking an important course [104].

*(2) Receptor for Advanced Glycation End Products (RAGE)*. A state of sustained hyperglycemia can promote protein and lipid glycation in consequence producing AGE, which promote the alteration of the structure and function of other proteins; AGE has affinity for receptors known as receptor for advanced glycation end product (RAGE). The RAGEs are immunoglobulins that when activated promote the secretion of cytokines, which further stimulate the complications of diabetes by increasing vascular permeability and inflammatory processes [105, 106]. These effects will promote a hypoxic state in the microcapillaries of the retina leading to the beginning of the angiogenic process in the PDR [106]. It has also been found that AGEs and RAGE are overexpressed in DR, which leads to think that genetic polymorphisms of RAGE are probably involved in the DR pathophysiology [107]. In a meta-analysis conducted by Yu et al. in 2016, they found that Gly82Ser in RAGE showed a significant association with DR; however, it was important to perform more studies with better control over the risk factors and duration of diabetes in patients [107]. There are other polymorphisms such as -429T/C, -374T/A, and 1704G/T, of which other studies have had contradictory results; therefore, without significant evidence, it has not been possible to associate them with DR [100, 105, 107].

*(3) Vascular Endothelial Growth Factor (VEGF)*. High levels of VEGF have been detected in the eyes of patients undergoing vitrectomy operations in patients with PDR; it is an important growth factor responsible for vascular permeability [103]. That is, high levels of VEGF promote a greater vascular permeability and neovascularization; therefore, it is said that the inhibition of this factor has shown an improvement of these events at the level of the retina. High levels of VEGF promote a greater vascular permeability and neovascularization, which is consistent with what several studies have shown, where patients with DR have a high expression of VEGF [103, 108]. Therefore, it is said that the inhibition of this factor has shown the improvement of these events at the level of the retina [103]. In a study conducted by Gonzalez-Salinas et al. [109] in the Mexican population, they aimed to associate the polymorphisms rs3025035, rs3025021, and rs2010963 that just increase the expression of VEGF and that were previously associated with PDR in other populations; however, their results did not allow them to create a significant association. It requires new studies with a larger sample size, knowledge about pharmacological treatment, and fewer restrictions on the patient's clinical information which is highlighted [109].

*(4) Nitric Oxide Synthase (NOS) Genes*. Nitric oxide has been detected in internal segments of photoreceptors, in some amacrine cells, in ganglion cells, and in the inner plexiform layer of the retina of adult rats [110]. The formation of NO is catalyzed by the enzyme endothelial nitric oxide synthase (eNOS) from L-arginine, which also takes a role in angiogenesis [97, 111, 112]. Therefore, eNOS is an important enzyme that contributes to vascular homeostasis in which overproduction can cause damage to the retina, by increased cell death, vascular permeability, and neurodegeneration mainly; eNOS polymorphisms have been related to increased risk to DR progress [110]. The decrement in the production of endothelial NOS can lead to the decrease of NO and vascular dilation [113].

Several analyzes have been made about the a/b polymorphism of the eNOS gene, and it has been argued that there is an association between this polymorphism and the risk of DR development [97, 112, 114]. A significant association was found between the intron 4a allele of the 4b/a polymorphism and a reduced risk of DR [114]. However, a meta-analysis indicates that the eNOS 4b/a polymorphism is not associated with an increased risk of DR among subjects with type 2 diabetes [97, 112].

As discussed here, many pathways and biomechanisms are implicated in DR; therefore, it is important to explore gene polymorphisms in enzymes and factors that play a role whether in redox balance, vascular function, or inflammation. Previously, we have discussed some of the most important polymorphisms linked to diabetic retinopathy; over the years, various studies have been done in this regard though results have been inconsistent. We present some examples of polymorphisms that have been associated with the diabetic retinopathy onset but have yet to be confirmed (see Table 1).

## 3. Influence of Antioxidants in Diabetic Retinopathy

Optimizing glycemic and lipid controls are the first-line therapies in diabetes control, which also reduce the DR progression [130]. Specific recommendations on diet as well as some of the dietary components or food intakes have already been reviewed on its effect on type 2 DM. Mediterranean diet is a recognized healthy dietary pattern [131] and has shown to have a protective effect against DR [131, 132] which contains a high amount of fish and extra virgin olive oil containing omega-3 fatty acids [133] and mixed nuts, which are rich on polyphenols that may reduce the risk of developing diabetes [134] and lowers insulin resistance [135]; also, it is rich in protective factors such as the Nrf2 [136] diet. Finally, the intake of vitamin-rich food such as fruits and vegetables as well as supplements has also been related to a risk reduction of chronic diseases [137] or DR itself [138], and they also have some hypoglycemic effects carried out by their bioactive compounds such as, flavonoids, alkaloids, and anthocyanins [139], the latter being present in wild blueberry, bilberry, cranberry, elderberry, raspberry seeds, and strawberry which have shown to have powerful antioxidant activity [140] while other micronutrients, such as vitamin C and E, have not shown any association between risk and intake [141] in contrast to Tanaka's prospective study on fruit consumption [142] but have yet to be explored on full potential in a possible combined-antioxidant therapy.

Nutrients in diet can play a massive role in diabetic patients who are resistant to conventional treatment; these nutritional strategies can reduce the risk of prognosis and attenuate progression preserving the normal function as well as structure of the retina [143].

As a complementary therapy to the existing conventional one, we propose the use of some supplements with antioxidant properties since they have protective effects at different points in the pathways involved in DR prognosis (see Figure 2).

### 3.1. Antioxidant Supplements

#### 3.1.1. Xanthophylls

Xanthophylls are natural pigments derived from carotenoids that contain oxygen. This family includes lutein and zeaxanthin. Both substances are found in the fovea; lutein concentration is superior to zeaxanthin which differs from lutein in its double link in one of the hydroxyl groups. They have antioxidant effect by alternating their single and double links reducing blue light wavelength and protecting the eye from light-induced oxidative stress. Around 90% of the blue light is absorbed by these pigments [144].

Astaxanthin is another xanthophyll which is extracted from H. pluvialis to be used as an alimentary supplement. According to a study, astaxanthin presents a larger biological activity compared to other antioxidants since it is able to bind both sides of the cell membrane [145].

According to various studies, lutein, xanthophylls, and other carotenoids have demonstrated to be useful in protecting the retina from OS in chronic hyperglycemic conditions and ameliorating oxidative stress states [146, 147]. Lutein quenches free radicals leading to the blockade of NF-*κ*B pathway activation and has effects on inflammation, by the inhibition of arachidonic acid release keeping prostaglandins, thromboxanes, and leukotrienes from being formed [144]. Lutein inhibits PI3K activity when it is increased secondary to oxidative stress via PI3K/Akt pathway which is capable of inhibiting the PDGF-induced RPE cell migration [148]. Lutein also is able to inhibit IL-8 secretion [149]; besides, zeaxanthin and lutein supplementation augments retinal pigment epithelial cell viability [150], and the former has been related to restoring VEGF concentrations [151]. Astaxanthin plays a role in the inhibition of proinflammatory molecule expression such as VEGF, ICAM-1, and MCP-1 [152]. In preclinical studies, astaxanthin has been shown to promote the expression of heme oxygenase-1 (HO-1) in the retina and greater glutamine synthase concentrations in Müller cells along with the reduction of H_2_O_2_-induced retinal ganglion cell apoptosis as well as improvement of MnSOD activity and decrement of oxidative damage markers [153, 154].

#### 3.1.2. Vitamin C

Vitamin C exists in two main forms, ascorbic and dehydroascorbic acid; it is a ubiquitous metabolite in plants and animals. Ascorbic acid acts as a cofactor alongside many human enzymes and as a water-soluble antioxidant [155]. Vitamin C is present in higher concentration in healthy patients, contrary to those with DR who have lower concentrations than those diabetic patients who have not developed this complication [156]. Vitamin C prevents the propagation of free radical-induced chain reactions [157], and thus, directly scavenging ROS preventing breakdown of NO and decreasing low-density lipid oxidation [143, 158, 159] protects the endothelial barrier permeability by the inhibition of VEFG [160]; however, caution is indeed needed since ascorbate can act as a prooxidant in the presence of transition metal such as ionic iron or ferritin, both associated with diabetes [161]. On the other hand, a supplementation with 1000 mg/day of ascorbic acid relates directly by reducing the activity of the enzyme aldose reductase and this way, it acts by inhibiting the polyol pathway [162]. Advanced glycation end products tend to decrease intracellular ascorbate; however, vitamin C also prevents the apoptosis of vascular pericytes [163]. It may have a role in autophagy, by the induction of autophagosome formation [164], increasing the rate of protein degradation lysosomes [165], and expressing Bcl-2 family proteins between hypoxia and reoxygenation statuses [166]. However, vitamin C has yet to be explored in DR since nothing similar has been reported in this diabetes complication.

#### 3.1.3. Vitamin E

Vitamin E is, contrary to vitamin C, a fat-soluble vitamin, and the predominant isomer found in human's body is alpha-tocopherol; because of this, it parts to lipid storage organelles and membranes [167]. Vitamin E has roles in many different explored mechanisms, one of them being on lipid peroxidation by inhibiting the formation of malondialdehyde [168, 169]; at concentrations as high as 2000 mg/day, it has been shown to reduce fasting plasma glucose in diabetes [159]. Also, the oxidative formation of N-epsilon-carboxymethyl-lysine in damaged proteins by long-term exposure to high-glucose concentration can be reduced by it [170]. Tocopherols can also modulate transduction and gene expression by modulating nuclear receptors for peroxisome proliferator-activated receptors [171]. Alpha-tocopherol at a concentration of 10 and 50 *μ*M was shown to inhibit smooth muscle proliferation as well as inhibit protein kinase C activity [172]. In a similar way, vitamin E has some effects on hemodynamic diabetes by decreasing the total diacylglycerol level, thus preventing the abnormal retinal flow [173]; furthermore, using the unsaturated vitamin E, tocotrienol, has an effect as an antiangiogenic agent by increasing apoptosis of signal-regulating kinase and p38 in the fibroblast growth factor [174]. As mentioned above, although vitamin E by itself has not proven its efficacy as a treatment for DR [175], more clinical studies are needed specially as a combined therapy, since it may have some more beneficial properties administered alongside other antioxidant compounds [176].

#### 3.1.4. Copper and Zinc

Zinc (Zn) is a nutritional element essential for the structure and function of numerous macromolecules, such as lipids, nucleic acids, and the enzymes, that regulate cellular processes and cellular signaling pathways [177]. Zn is widely distributed in foods and beverages, but as with other elements, the contents are variable and generally low [178].

Zinc exhibits antioxidant and anti-inflammatory activities, delaying oxidative processes in the long term by inducing the expression of metallothioneins (MT), and acts as a cofactor of the cytosolic and extracellular Zn/Cu SOD enzyme, which scavenges ROS by catalyzing the dissociation of the O_2_^−^ radical in the less harmful forms O_2_ and H_2_O_2_ [177]. Copper (Cu) participates in the production of energy in the mitochondria and functions as a cofactor to superoxide dismutase (SOD) found in the cytosol and intracellular space. Over the years, copper imbalances have been linked to chronic inflammatory diseases [179].

Oxidative stress (OS) influences the molecular mechanisms responsible for the development of many inflammatory diseases, such as DM [177]. It has been shown that zinc supplementation is beneficial for the balance between the content of free radicals and antioxidant enzyme systems in rats with systematic inflammatory response [180]. It is possible that these supplements improve the absorption in food of vitamin E and therefore prevent deficiency [181].

Zn supplementation increases insulin sensitivity and antioxidant capacity [182]. In these models in which diabetes was induced, the antioxidant enzymes catalase, GPx (glutathione peroxidase), and superoxide dismutase (SOD) are diminished in comparison with normal animals. Zn supplementation in these animals restored the activity of the enzyme and the synthesis of glutathione [182] and also attenuates the OS induced by diabetes in the circulation, as well as in cardiac and hepatic tissues in diabetic rats [183]. Renal oxidative damage induced by diabetes and inflammation has been significantly attenuated by Zn supplementation, mediated through MT expression [182]. Regarding the metabolism of glucose and lipids, the blood glucose level is also reduced in type 2 diabetic rats given with ZnO nanoparticles, with better glucose tolerance and a 70% increase in insulin levels. In addition to the significant reduction of circulating triglycerides and free fatty acids [184], Zn deficiency can have serious implications on the elderly; therefore, it is important to maintain adequate nutrition of Zn in this population [185].

It is known that more than 100 specific enzymes require Zn for their catalytic function, which indicates the critical role of Zn in cellular processes [186], including events of genomic stability, cognitive functions, depression, and oxidative stress [185]. Zinc alone is not actively redox, and therefore, Zn^2+^ does not interact directly with ROS or with free radicals centered on carbon [187, 188]. Zinc then contributes to the antioxidant status through its ability to compete with transition metals and copper for binding sites in the cell membrane [183]. Iron and copper ions catalyze the production of lipoperoxides; therefore, their replacement by zinc under conditions of insulin resistance in the plasma membrane could inhibit lipoperoxides [189]. Several studies in animals and humans have found that high levels of supplemental Zn over long periods of time can result in a decrease in the absorption of Cu leading to Cu deficiency [190].

#### 3.1.5. Alpha Lipoic Acid

Alpha lipoic acid also called thioctic acid is a natural compound found primarily in vegetables (broccoli, spinach, and tomatoes) and meats and nowadays in many additives. Alpha lipoic is both hydrophilic and hydrophobic and widely distributed both in cellular membranes and the cytosol and is essential for mitochondrial function [191]. It has been named as the “universal antioxidant” [192] since once consumed it is reduced to dihydrolipoic acid and both lipoic and dihydrolipoic acid can inhibit lipid and protein oxidation, as well as ROS scavengers [193]; not only that, lipoic acid also induced Nrf2 binding to antioxidant response elements and thus higher gamma glutamylcysteine ligase and its catalytic subunit, and this way, it ameliorates this antioxidant loss related with age [194]. Finally and importantly, as of why combined antioxidant therapies are not only viable but also synergized, dihydrolipoic acid can regenerate endogenous antioxidants, particularly vitamins C and E, two of the revised antioxidants in this article, and glutathione [195]. Alpha lipoic acid has antiangiogenic activity; it has proven to be effective in reducing the VEGF, angiopoietin 2, and erythropoietin by blocking superoxide formation in diabetic rat's retina [196] and by protecting the retinal ganglion cells by preserving its thickness [197], but it also has a direct antiangiogenic role, by inhibiting endothelial cell apoptosis and proliferation (not related to pericytes) through a probable inhibition of NF-*κ*B, activating protein kinase B and upregulating p27 activity (inhibiting cell cycle progression) [198]. Alpha lipoic acid has even been formulated as an aqueous solution and administered intravenously, intraperitoneally, and intravitreally to evaluate the activity on microvascular complications in the eye by fluorescein leakage and by direct observation that concluded to reduce these complications and slow the progression of diabetic retinopathy [199]. Alpha lipoic acid's beneficial properties were also assessed on mitochondrial metabolism; in one study, mitochondrial function and regulation, measured by its transcriptional factor, peroxisome proliferator-activated receptor-*γ* coactivator-1*α*, and nuclear respiratory factor 1 was benefited by lipoic acid by preventing the loss of the mitochondrial copy number and increasing gene transcripts of PPAR*γ* and NRF1 [200]. Some preclinical studies have shown efficacy of lipoic acid therapy in DR [201, 202], and clinically, it may have a protective role [203] but has yet to show efficacy on patients who have already developed DR, as it has shown no effect on macular edema at a daily dose of 600 mg [204].

#### 3.1.6. Manganese

Manganese (Mn) is a heavy metal present in nature and is the fifth most abundant metal in the environment. Mn is essential for humans and animals; daily requirements are usually met with a proper diet. High levels of manganese can be found in legumes, rice, nuts, and whole grains [205]. Mn is transported by simple diffusion in the large intestine and is absorbed by active transport in the small intestine. Only about 5% of the Mn in the diet seems to be absorbed [206]. Mn is involved in cellular antioxidant defense mechanisms, but it is known that it participates in the generation of ROS and has prooxidative properties [207]. Mn is an essential nutrient that is required as part of a healthy diet; however, exposure to excessive levels results in toxicity in human development leading to hyperactivity, inferior intellectual function, impaired motor skills, and reduced olfactory function in children [205].

Mn is a cofactor in the key mitochondrial antioxidant enzyme [207] and a component of metalloenzymes such as Mn superoxide dismutase (MnSOD), glutamate synthetase, and pyruvate carboxylase and is associated with oxidative phosphorylation and mucopolysaccharide metabolism [206]. MnSOD main function is the detoxification of superoxide free radicals [208]. Mn can provide resistance to oxidative stress through the formation of manganese-based nonprotein antioxidants and also function safely as a cofactor for the enzyme superoxide dismutase (SOD) [209]. Given the similar physical properties between Fe and Mn, most transporters are capable of transporting both metals, which are competent to bind to the plasma membrane [205]. Several proteins involved in the transport of Mn have been identified, including the putative uptake proteins divalent metal transporter-1 (DMT1), transferrin receptor (TfR), and ATP13A2, as well as the efflux protein Fpn [206].

It was found that Mn is required for synthesis and secretion of normal insulin from an initial study in rats. Rats that are on a high-fat diet can improve glucose tolerance and insulin secretion. The fact that Mn results in insulin secretion induced by glucose is consistent with the improvement of mitochondrial function with glucose metabolism [210].

#### 3.1.7. Curcumin

Curcumin is one of the main substances of *Curcuma* spp.; it is a crystalline orange-yellow color compound [211]. World Health Organization (WHO) recommended a minimal diary intake of 0-3 mg/kg as a food additive [212]. In recent years, it has been shown that curcumin has beneficial properties in DR treatment. (1) Curcumin acts as an antioxidant agent by reducing free radicals [213]. (2) Curcumin increases mRNA expression of antioxidant enzymes like SOD and catalase by reducing oxidative and regulating nitrosative DNA damage [214]. (3) Curcumin activates a mitochondrial pathway by regulating the respiratory function on mitochondrial complexes I, II, III, and V and simultaneously activates Nrf2 [215]. (4) Curcumin can increase antioxidant capacity in the retina of diabetic rats and hypoglycemic and preventive anti-inflammatory activity by reducing the levels of proinflammatory cytokines like IL-1*β*, tumor necrosis factor alpha, VEGF [216, 217], and 5-hydroxyeicosatetraenoic acid being a dual inhibitor of arachidonic acid [218]. (5) Curcumin acts as an antiangiogenic agent by decreasing stromal cell-derived factor 1 alpha that inhibits the migration of retinal human endothelial cells [219].

As seen in Figure 2, curcumin induces Nrf2 pathway activation, helping into a better defense against oxidative stress in retinal cells [220, 221]. All these effects related to diabetes and more specifically DR on curcumin are a promising alternative for the treatment of DR [222, 223]. Attention is needed in the presence of high concentrations since curcumin can act as a prooxidant agent and induce apoptosis [224].

#### 3.1.8. Anthocyanins

Anthocyanins belong to the flavonoid group; they are six polyphenolic pigments in which 90% of the composition are found in nature: pelargonidin, cyaniding, peonidin, delphinidin, petunidin, and malvidin [225] while in the body, they are mostly metabolized to phenolic acid and degradation products and are a stable water-soluble compounds [226], and they can be found deposited in the eye [227]. These compounds have been studied recently and extensively, and their effects are primarily on cardiovascular diseases; here, we try to summarize those related to DR pathogenesis. Anthocyanins alongside other bioactive compounds have a role as antioxidants by scavenging ROS [228], by inhibiting lipid peroxidation [229] and induction of Nrf2 expression (see Figure 2) [230], and by their antioxidant properties; anthocyanins induce the downregulation of the NF-*κ*B signaling pathway exerting an anti-inflammatory response, and it may be as well partially involved in the mitogen-activated protein kinase pathways [231, 232]. Given all of these beneficial effects, more clinical interventions are needed to prove or assess these effects on diabetic retinopathy, rather than diabetes itself [233].

#### 3.1.9. Ubiquinone

Ubiquinone or coenzyme Q10 (CoQ10) is ubiquitous in nature and widely distributed in plants, animals, and microorganisms. Ubiquinone can be obtained through exogenous sources, such as food. The richest dietary sources are meat, migratory fish, some oils, and nuts, but in the diet of the populations of western countries, these sources contribute in total to only 3-5 mg of CoQ10 per day [234]. A dose that varies from 50 to 150 mg is recommended in food supplements; however, there are also products with higher levels available [234]. In diabetes, the resulted hyperglycemia state induces the overproduction of superoxide by the electron transport chain in the mitochondria; this leads to vascular damage mediated by glucose [235].

Coenzyme Q10 (CoQ10) or ubiquinone is an essential compound found naturally in all cells of the human body. It is particularly known for its role in the chain of electron transport in mitochondrial membranes during aerobic cellular respiration. It is the only lipid-soluble antioxidant that animal cells synthesize de novo in the body [236] and is able to recycle and regenerate other antioxidants such as tocopherol and ascorbate [235]. Coenzyme Q10 is part of the process of oxidative phosphorylation in mitochondria, where it converts energy into carbohydrates and fatty acids into ATP to boost cellular machinery and synthesis. In addition to facilitating the transfer of electrons during oxidative phosphorylation, CoQ10 acts by inhibiting certain enzymes involved in the formation of free radicals, thereby reducing the consequences of oxidative stress [237].

One of the most important mechanisms offered by coenzyme Q10 to protect against diabetes is through the “recoupling” of the endothelial NOS. Increased oxidative stress in diabetes can cause diabetics to reduce the biological availability of nitric oxide [238]. Coenzyme Q10 acts by blocking endothelial dysfunction by activating endothelial nitric oxide synthase and mitochondrial oxidative phosphorylation. Thus, supplementation with coenzyme Q10 shown to alleviate the symptoms in animals and humans, by decreasing blood pressure in hypertensive individuals [238]. The treatment with CoQ10 presented several benefits, among them are the significant decrease in the high levels of glucose, triglycerides, very low-density lipoproteins, low-density lipoproteins, and atherogenic index and increase in the levels of high-density lipoproteins in diabetic rats. It also reduced lipid peroxidation and increased antioxidant parameters such as superoxide dismutase, catalase, and glutathione in the homogenates of diabetic rats [237].

#### 3.1.10. Resveratrol

Resveratrol (3,5,4′-trihydroxystilbene (RSV)) is a natural phenol produced by several plants in response to damage when the plant is under attack by microorganisms. RSV is found in red wine and the skin of grapes, but also in blueberries, raspberries, and mulberries. RSV has antiproliferative, antiangiogenic, antioxidant, endothelial, anti-inflammatory, antiplatelet, and neurogenic activity [239, 240]. Resveratrol exists in both *cis* and *trans* form, and it is believed that the *trans* form is more stable [240]. RSV is absorbed by 75%, mainly by transepithelial diffusion, but when taken orally, bioavailability is very low, less than 1%; this is because in the intestine and liver, the metabolism is rapid and glucuronidated compounds are involved and sulfated to generate key metabolites that are easily eliminated. However, bioavailability is very variable between one individual and another due to factors such as age and gender [240–242]. As RSVl is a hydrophobic compound, it has been shown to be absorbed by intestinal epithelial cells, hepatocytes, and breast tumor cell lines [240]. It was found that the treatment with resveratrol causes an increase in the levels of reduced glutathione (GSH) in erythrocytes and the ocular level in rats, where GSH has a protective function against oxidants; also, significantly lower concentrations of malondialdehyde were found, which is a marker of peroxidation lipid [243, 244]. RSV also suppresses the action of endothelial nitric oxide synthase in the eyes of rats, an enzyme associated with neovascularization and with inflammatory processes in diabetes. Resveratrol use as a treatment creates a beneficial effect on the increase in vascular leaks, in the loss of pericytes, and the levels of VEGF [245, 246]. A study conducted by Luna et al. [247] showed that resveratrol also inhibited the production of reactive oxygen species (ROS), which in turn prevented the induction of proinflammatory markers such as interleukin-1a (IL-1a), interleyukin-6 (IL-6), and interleukin-8 (IL-8).

#### 3.1.11. Omega-3

Lipids are important for cellular signals and metabolism, since they are part of the structure of the membranes and storage energy, so lipids and their metabolites are of great importance in ocular diseases because they are regulators in neovascularization [248]. The omega-3 are a family of healthy fats and are within the monounsaturated and polyunsaturated fatty acids. They are obtained from marine sources and also have anti-inflammatory and antiangiogenic properties which have been investigated in various parts of the human body, including the retina. There are three types of omega-3 fatty acids: alpha-linoleic acid (ALA), eicosapentaenoic acid (EPA), and docosahexaenoic acid (DHA) [249]. Because the retina is a tissue with high lipid content, it receives high amounts of oxygen, so it is highly vulnerable to oxidative stress; reactive oxygen species carry out lipid peroxidation causing damage to membranes, proteins, and the nuclear DNA. It is also known that the deficient consumption of omega-3 contributes to the degeneration of the retina [250]. In the trial, PREDIMED (Prevention with Mediterranean Diet), which followed a 6-year follow-up of middle-aged and older individuals with diabetes mellitus type 2 with adherence to a “healthy” Mediterranean diet and demonstrated a subset of patients whose diet includes omega-3 polyunsaturated fatty acids, show a 48% decreased incidence in DR [248, 251]. Both hyperglycemia and dyslipidemias are associated with DR, and although a strict diet control could delay the onset of retinopathy in patients with T2DM, this is not always achieved; however, in a trial, it was shown that a diet rich in foods with omega-3, similar to a Japanese diet, effectively reduced pathological neovascularization in the retina when compared to a diet rich in omega-6, apparently similar to an American diet [252]. Given that current treatments to counteract DR are costly and generally invasive, nutritional interventions have the potential to significantly improve microvascular complications resulting from diabetes. For this, diets rich in omega-3 can diminish the visual deterioration that appears in the first stages of the DR in a safe and accessible long before clinical manifestations [251].

## 4. Conclusion

At the beginning, there was a debate whether diabetic retinopathy was mainly a neuropathy or a vasculopathy. Through years of investigation, neural damage have shown to occur before vascular changes in the retinal tissue. Nevertheless, both have similitudes in the mechanisms involved and are present at different stages of the disease, and they continue; at the same time, hyperglycemia leads to inflammatory response causing cellular degeneration, endothelial insult, and hypoxia which in turn leads to more inflammatory response. At the same time, hyperglycemia induces ROS generation. Nevertheless, it is known that lowering glucose levels in diabetic patients remains the best way to avoid complications from diabetes as many studies have shown; however, this goal is hard to achieve for many patients; for that reason, we propose a multitarget therapy including oxidative stress-lowering strategies. Studies have demonstrated that oxidative stress plays an important role in all the described mechanisms by enhancing inflammatory responses, mediating the expression of prodegenerative and proinflammatory proteins, causing damage in cellular structures and functions. Genetic alterations involving antioxidant defenses are found to be linked to DR worsening or speeding up the onset, supporting the importance of oxidative stress as a pillar of diabetic retinopathy pathophysiology thus endorsing antioxidant supplementation as an adjuvant therapy along with diabetes management.

## Figures and Tables

**Table 1:** Polymorphisms implicated in diabetic retinopathy. Many genes have been associated with diabetic retinopathy; some polymorphisms in them have, apparently, protective effects while others worsen its progression.

	Author (year)	Population	Polymorphism	Conclusions
Aldose reductase (Alr)	Abhary et.al. (2010) [102]	Australian	rs9640883	Association with duration of diabetes rather than a direct association to DR
	Wang et al. (2003) [115]	Chinese	Rs759853 T allele	Protective effect against DR in DM type 1
	Santos et al. (2003) [116]	Euro-Brazilian	ALR C(-106)T	No association to DR
Nitric oxide synthase (NOS)	Zhao et al. (2012) [114]	Chinese	NOS3 4b/a	Negative association with DR (protective effect)
	Cheema et al. (2012) [117]	Asian Indian	rs3138808	No association with DR
	Santos et al. (2012) [118]	Caucasian-Brazilian	NOS3b/a	No association to DR
Receptor for advanced glycation end products (RAGEs)	Ng et al. (2012) [119]	Malaysian	-429T/C and -374T/A	No association with DR
	Vanita (2014) [120]	Indian	Gly82Ser	Positive association with DR
	Yang et al. (2013) [121]	Chinese	Gly82Ser	Associated to DR risk
Vascular endothelial growth factor (VEGF)	Kangas-Kontio et al. (2009) [122]	Multiethnic	rs3095039	No association
	Abhary et al. (2009) [123]	Multiethnic	rs3025021	Positive association
	Qiu et al. (2013) [124]	Chinese	rs2010963	Positive association
Gluthatione S-transferase (GST)	Dadbinpour et al. (2013) [125]	Iranian	GSTM1	Positive association with DR
Manganese superoxide dismutase (MnSOD)	Haghighi et al. (2015) [126]	Iranian	A16V	Positive association with DR
	Vanita (2014) [120]	Indian	Val16Ala	No association with DR
Intercellular adhesion molecule1 (ICAM-1)	Fan et al. (2015) [127]	Asian	rs5498	Negative association with DR
			Rs13306430	Positive association with DR
Transforming growth factor beta 1 (TGF-*β*1)	Rodrigues et al. (2015) [128]	Brazilian	Rs1800471	Positive association with DR
	Bazzaz et al. (2014) [129]	Caucasian	+869 C/T +915 G/C	No association with DR

## References

[1] Shaw J. E., Sicree R. A., Zimmet P. Z. (2010). Global estimates of the prevalence of diabetes for 2010 and 2030. *Diabetes Research and Clinical Practice*.

[2] Yuan T., Yang T., Chen H. (2019). New insights into oxidative stress and inflammation during diabetes mellitus-accelerated atherosclerosis. *Redox Biology*.

[3] Zheng Y., He M., Congdon N. (2012). The worldwide epidemic of diabetic retinopathy. *Indian Journal of Ophthalmology*.

[4] Distefano L. N., Garcia-Arumi J., Martinez-Castillo V., Boixadera A. (2017). Combination of anti-VEGF and laser photocoagulation for diabetic macular edema: a review. *Journal of Ophthalmology*.

[5] Claramunt J. (2009). Diabetic retinopathy. *Revista Médica Clínica Las Condes*.

[6] Koopman W. J. H., Nijtmans L. G. J., Dieteren C. E. J. (2010). Mammalian mitochondrial complex I: biogenesis, regulation, and reactive oxygen species generation. *Antioxidants & Redox Signaling*.

[7] Park S., Kang H. J., Jeon J. H., Kim M. J., Lee I. K. (2019). Recent advances in the pathogenesis of microvascular complications in diabetes. *Archives of Pharmacal Research*.

[8] Barrett E. J., Liu Z., Khamaisi M. (2017). Diabetic microvascular disease: an Endocrine Society scientific statement. *The Journal of Clinical Endocrinology & Metabolism*.

[9] Aghadavod E., Khodadadi S., Baradaran A., Nasri P., Bahmani M., Rafieian-Kopaei M. (2016). Role of oxidative stress and inflammatory factors in diabetic kidney disease. *Iranian Journal of Kidney Diseases*.

[10] Jha J. C., Banal C., Chow B. S. M., Cooper M. E., Jandeleit-Dahm K. (2016). Diabetes and kidney disease: role of oxidative stress. *Antioxidants & Redox Signaling*.

[11] Sifuentes-Franco S., Pacheco-Moisés F. P., Rodríguez-Carrizalez A. D., Miranda-Díaz A. G. (2017). The role of oxidative stress, mitochondrial function, and autophagy in diabetic polyneuropathy. *Journal of Diabetes Research*.

[12] Weng L., Zhang F., Wang R., Ma W., Song Y. (2019). A review on protective role of genistein against oxidative stress in diabetes and related complications. *Chemico-Biological Interactions*.

[13] Bao L., Li J., Zha D. (2018). Chlorogenic acid prevents diabetic nephropathy by inhibiting oxidative stress and inflammation through modulation of the Nrf2/HO-1 and NF-*ĸ*B pathways. *International Immunopharmacology*.

[14] Rossino M. G., Casini G. (2019). Nutraceuticals for the treatment of diabetic retinopathy. *Nutrients*.

[15] Laddha A. P., Kulkarni Y. A. (2019). Tannins and vascular complications of diabetes: an update. *Phytomedicine*.

[16] Whitehead M., Wickremasinghe S., Osborne A., van Wijngaarden P., Martin K. R. (2018). Diabetic retinopathy: a complex pathophysiology requiring novel therapeutic strategies. *Expert Opinion on Biological Therapy*.

[17] Lehninger A., Nelson D. (2000). *Lehninger Principles of Biochemistry*.

[18] Heng L. Z., Comyn O., Peto T. (2013). Diabetic retinopathy: pathogenesis, clinical grading, management and future developments. *Diabetic Medicine*.

[19] Behl T., Kotwani A. (2015). Exploring the various aspects of the pathological role of vascular endothelial growth factor (VEGF) in diabetic retinopathy. *Pharmacological Research*.

[20] Phillips S. A., Thornalley P. J. (1993). The formation of methylglyoxal from triose phosphates: Investigation using a specific assay for methylglyoxal. *European Journal of Biochemistry*.

[21] Schlotterer A., Kolibabka M., Lin J. (2019). Methylglyoxal induces retinopathy-type lesions in the absence of hyperglycemia: studies in a rat model. *The FASEB Journal*.

[22] Akimoto Y., Kreppel L. K., Hirano H., Hart G. W. (2001). Hyperglycemia and the O-GlcNAc transferase in rat aortic smooth muscle cells: elevated expression and altered patterns of O-GlcNAcylation. *Archives of Biochemistry and Biophysics*.

[23] dela Justina V., Gonçalves J. S., de Freitas R. A. (2017). Increased *O*-linked N-acetylglucosamine modification of NF-*ĸ*B and augmented cytokine production in the placentas from hyperglycemic rats. *Inflammation*.

[24] Choudhuri S., Chowdhury I. H., Das S. (2015). Role of NF-*κ*B activation and VEGF gene polymorphisms in VEGF up regulation in non-proliferative and proliferative diabetic retinopathy. *Molecular and Cellular Biochemistry*.

[25] Murakami T., Felinski E. A., Antonetti D. A. (2009). Occludin phosphorylation and ubiquitination regulate tight junction trafficking and vascular endothelial growth factor-induced permeability. *Journal of Biological Chemistry*.

[26] Hendrick A. M., Gibson M. V., Kulshreshtha A. (2015). Diabetic retinopathy. *Primary Care: Clinics in Office Practice*.

[27] Devi T. S., Lee I., Hüttemann M., Kumar A., Nantwi K. D., Singh L. P. (2012). TXNIP links innate host defense mechanisms to oxidative stress and inflammation in retinal Muller glia under chronic hyperglycemia: implications for diabetic retinopathy. *Experimental Diabetes Research*.

[28] Bain S. C., Klufas M. A., Ho A., Matthews D. R. (2019). Worsening of diabetic retinopathy with rapid improvement in systemic glucose control: a review. *Diabetes, Obesity and Metabolism*.

[29] Ahsan H. (2015). Diabetic retinopathy – biomolecules and multiple pathophysiology. *Diabetes & Metabolic Syndrome: Clinical Research & Reviews*.

[30] Packer L., Cadenas E. (2007). Oxidants and antioxidants revisited. New concepts of oxidative stress. *Free Radical Research*.

[31] Miyamoto N., de Kozak Y., Jeanny J. C. (2007). Placental growth factor-1 and epithelial haemato–retinal barrier breakdown: potential implication in the pathogenesis of diabetic retinopathy. *Diabetologia*.

[32] Brownlee M. (2001). Biochemistry and molecular cell biology of diabetic complications. *Nature*.

[33] Giacco F., Brownlee M. (2010). Oxidative stress and diabetic complications. *Circulation Research*.

[34] Yan L.-J. (2018). Redox imbalance stress in diabetes mellitus: role of the polyol pathway. *Animal Models and Experimental Medicine*.

[35] Aldieri E., Riganti C., Polimeni M. (2008). Classical inhibitors of NOX NAD(P)H oxidases are not specific. *Current Drug Metabolism*.

[36] Cumaoǧlu A., Cevik Ç. E., Rackova L., Ari N., Karasu Ç. I. (2007). Effects of antioxidant stobadine on protein carbonylation, advanced oxidation protein products and reductive capacity of liver in streptozotocin-diabetic rats: role of oxidative/nitrosative stress. *Biofactors*.

[37] Gomes A. P., Price N. L., Ling A. J. Y. (2013). Declining NAD^+^ induces a pseudohypoxic state disrupting nuclear-mitochondrial communication during aging. *Cell*.

[38] Ido Y., Williamson J. R. (1997). Hyperglycemic cytosolic reductive stress ‘pseudohypoxia’: implications for diabetic retinopathy. *Investigative Ophthalmology & Visual Science*.

[39] Chung S. S. M., Ho E. C. M., Lam K. S. L., Chung S. K. (2003). Contribution of polyol pathway to diabetes-induced oxidative stress. *Journal of the American Society of Nephrology*.

[40] Gugliucci A. (2017). Formation of fructose-mediated advanced glycation end products and their roles in metabolic and inflammatory diseases. *Advances in Nutrition: An International Review Journal*.

[41] Saxena R., Singh D., Saklani R., Gupta S. K. (2016). Clinical biomarkers and molecular basis for optimized treatment of diabetic retinopathy: current status and future prospects. *Eye and Brain*.

[42] Obrosova I., Cao X., Greene D. A., Stevens M. J. (1998). Diabetes-induced changes in lens antioxidant status, glucose utilization and energy metabolism: effect of DL-*α*-lipoic acid. *Diabetologia*.

[43] Jedziniak J. A., Chylack L. T., Cheng H. M., Gillis M. K., Kalustian A. A., Tung W. H. (1981). The sorbitol pathway in the human lens: aldose reductase and polyol dehydrogenase. *Investigative Ophthalmology & Visual Science*.

[44] Srinivasan V., Sandhya N., Sampathkumar R., Farooq S., Mohan V., Balasubramanyam M. (2007). Glutamine fructose-6-phosphate amidotransferase (GFAT) gene expression and activity in patients with type 2 diabetes: inter-relationships with hyperglycaemia and oxidative stress. *Clinical Biochemistry*.

[45] Das Evcimen N., King G. (2007). The role of protein kinase C activation and the vascular complications of diabetes. *Pharmacological Research*.

[46] Liu Z. C., Yu E. H., Liu W., Liu X. C., Tang S. B., Zhu B. H. (2014). Translocation of protein kinase C *δ* contributes to the moderately high glucose-, but not hypoxia-induced proliferation in primary cultured human retinal endothelial cells. *Molecular Medicine Reports*.

[47] Jiang Y., Zhang Q., Steinle J. J. (2015). Beta-adrenergic receptor agonist decreases VEGF levels through altered eNOS and PKC signaling in diabetic retina. *Growth Factors*.

[48] Dekker L. V., Leitges M., Altschuler G. (2000). Protein kinase C-*β* contributes to NADPH oxidase activation in neutrophils. *Biochemical Journal*.

[49] Lei S., Su W., Liu H. (2013). Nitroglycerine-induced nitrate tolerance compromises propofol protection of the endothelial cells against TNF-*α*: the role of PKC-*β*_2_ and NADPH oxidase. *Oxidative Medicine and Cellular Longevity*.

[50] Byun H. O., Jung H. J., Kim M. J., Yoon G. (2014). PKC*δ* phosphorylation is an upstream event of GSK3 inactivation-mediated ROS generation in TGF-*β*1-induced senescence. *Free Radical Research*.

[51] Ramasamy R., Shekhtman A., Schmidt A. M. (2016). The multiple faces of RAGE – opportunities for therapeutic intervention in aging and chronic disease. *Expert Opinion on Therapeutic Targets*.

[52] Homme R. P., Singh M., Majumder A. (2018). Remodeling of retinal architecture in diabetic retinopathy: disruption of ocular physiology and visual functions by inflammatory gene products and pyroptosis. *Frontiers in Physiology*.

[53] Lukiw W. J., Ottlecz A., Lambrou G. (2003). Coordinate activation of HIF-1 and NF-*κ*B DNA binding and COX-2 and VEGF expression in retinal cells by hypoxia. *Investigative Opthalmology & Visual Science*.

[54] Hsiao K. Y., Lin S. C., Wu M. H., Tsai S. J. (2015). Pathological functions of hypoxia in endometriosis. *Frontiers in Bioscience*.

[55] Cheng J., Yang H.‑. L., Gu C.‑. J. (2019). Melatonin restricts the viability and angiogenesis of vascular endothelial cells by suppressing HIF-1*α*/ROS/VEGF. *International Journal of Molecular Medicine*.

[56] Liang X., Zhang D., Liu W. (2017). Reactive oxygen species trigger NF-*κ*B-mediated NLRP3 inflammasome activation induced by zinc oxide nanoparticles in A549 cells. *Toxicology and Industrial Health*.

[57] El-Remessy A. B., Al-Shabrawey M., Khalifa Y., Tsai N.-T., Caldwell R. B., Liou G. I. (2006). Neuroprotective and blood-retinal barrier-preserving effects of cannabidiol in experimental diabetes. *The American Journal of Pathology*.

[58] Yerramothu P., Vijay A. K., Willcox M. D. P. (2018). Inflammasomes, the eye and anti-inflammasome therapy. *Eye*.

[59] Feenstra D. J., Yego E. C., Mohr S. (2013). Modes of retinal cell death in diabetic retinopathy. *Journal of Clinical & Experimental Ophthalmology*.

[60] Akhtar-Schäfer I., Wang L., Krohne T. U., Xu H., Langmann T. (2018). Modulation of three key innate immune pathways for the most common retinal degenerative diseases. *EMBO Molecular Medicine*.

[61] Di Rosa M., Distefano G., Gagliano C., Rusciano D., Malaguarnera L. (2016). Autophagy in diabetic retinopathy. *Current Neuropharmacology*.

[62] Kowluru R. A., Zhong Q., Santos J. M. (2012). Matrix metalloproteinases in diabetic retinopathy: potential role of MMP-9. *Expert Opinion on Investigational Drugs*.

[63] Yang J. S., Lu C. C., Kuo S. C. (2017). Autophagy and its link to type II diabetes mellitus. *BioMedicine*.

[64] White E., Mehnert J. M., Chan C. S. (2015). Autophagy, metabolism, and cancer. *Clinical Cancer Research*.

[65] Parzych K. R., Klionsky D. J. (2014). An overview of autophagy: morphology, mechanism, and regulation. *Antioxidants & Redox Signaling*.

[66] Sarparanta J., Garcia-Macia M., Singh R. (2017). Autophagy and mitochondria in obesity and type 2 diabetes. *Current Diabetes Reviews*.

[67] Frost L. S., Mitchell C. H., Boesze-Battaglia K. (2014). Autophagy in the eye: implications for ocular cell health. *Experimental Eye Research*.

[68] Demirtas L., Guclu A., Erdur F. M. (2016). Apoptosis, autophagy & endoplasmic reticulum stress in diabetes mellitus. *The Indian Journal of Medical Research*.

[69] Cachafeiro M., Bemelmans A. P., Samardzija M. (2013). Hyperactivation of retina by light in mice leads to photoreceptor cell death mediated by VEGF and retinal pigment epithelium permeability. *Cell Death & Disease*.

[70] Jain A., Saxena S., Khanna V. K., Shukla R. K., Meyer C. H. (2013). Status of serum VEGF and ICAM-1 and its association with external limiting membrane and inner segment-outer segment junction disruption in type 2 diabetes mellitus. *Molecular Vision*.

[71] Joussen A. M., Poulaki V., Qin W. (2002). Retinal vascular endothelial growth factor induces intercellular adhesion molecule-1 and endothelial nitric oxide synthase expression and initiates early diabetic retinal leukocyte adhesion *in vivo*. *The American Journal of Pathology*.

[72] Shin H. J., Lee S. H., Chung H., Kim H. C. (2012). Association between photoreceptor integrity and visual outcome in diabetic macular edema. *Graefe's Archive for Clinical and Experimental Ophthalmology*.

[73] Meleth A. D., Agro´n E., Chan C. C. (2005). Serum inflammatory markers in diabetic retinopathy. *Investigative Opthalmology & Visual Science*.

[74] Goldstein I. M., Ostwald P., Roth S. (1996). Nitric oxide: a review of its role in retinal function and disease. *Vision Research*.

[75] Sato M., Ohtsuka T., Stell W. K. (2011). Endogenous nitric oxide enhances the light-response of cones during light-adaptation in the rat retina. *Vision Research*.

[76] Chakravarthy H., Devanathan V. (2018). Molecular mechanisms mediating diabetic retinal neurodegeneration: potential research avenues and therapeutic targets. *Journal of Molecular Neuroscience*.

[77] Kim K., Kim E. S., Yu S. Y. (2018). Longitudinal relationship between retinal diabetic neurodegeneration and progression of diabetic retinopathy in patients with type 2 diabetes. *American Journal of Ophthalmology*.

[78] Abcouwer S. F., Gardner T. W. (2014). Diabetic retinopathy: loss of neuroretinal adaptation to the diabetic metabolic environment. *Annals of the New York Academy of Sciences*.

[79] Kern T. S., Tang J., Berkowitz B. A. (2010). Validation of structural and functional lesions of diabetic retinopathy in mice. *Molecular Vision*.

[80] Lynch S. K., Abramoff M. D. (2017). Diabetic retinopathy is a neurodegenerative disorder. *Vision Research*.

[81] Ly A., Yee P., Vessey K. A., Phipps J. A., Jobling A. I., Fletcher E. L. (2011). Early inner retinal astrocyte dysfunction during diabetes and development of hypoxia, retinal stress, and neuronal functional loss. *Investigative Opthalmology & Visual Science*.

[82] Kalesnykas G., Tuulos T., Uusitalo H., Jolkkonen J. (2008). Neurodegeneration and cellular stress in the retina and optic nerve in rat cerebral ischemia and hypoperfusion models. *Neuroscience*.

[83] Semeraro F., Cancarini A., dell’Omo R., Rezzola S., Romano M. R., Costagliola C. (2015). Diabetic retinopathy: vascular and inflammatory disease. *Journal of Diabetes Research*.

[84] Arjamaa O., Aaltonen V., Piippo N. (2017). Hypoxia and inflammation in the release of VEGF and interleukins from human retinal pigment epithelial cells. *Graefe's Archive for Clinical and Experimental Ophthalmology*.

[85] Wang S., Ji L.‑. Y., Li L., Li J.‑. M. (2018). Oxidative stress, autophagy and pyroptosis in the neovascularization of oxygen‑induced retinopathy in mice. *Molecular Medicine Reports*.

[86] Yan X., Tezel G., Wax M. B., Edward D. P. (2000). Matrix metalloproteinases and tumor necrosis factor *α* in glaucomatous optic nerve head. *Archives of Ophthalmology*.

[87] Wakatsuki S., Furuno A., Ohshima M., Araki T. (2015). Oxidative stress-dependent phosphorylation activates ZNRF1 to induce neuronal/axonal degeneration. *The Journal of Cell Biology*.

[88] Wang J., He C., Zhou T., Huang Z., Zhou L., Liu X. (2016). NGF increases VEGF expression and promotes cell proliferation via ERK1/2 and AKT signaling in Müller cells. *Molecular Vision*.

[89] Sun Z., Hu W., Yin S. (2017). NGF protects against oxygen and glucose deprivation-induced oxidative stress and apoptosis by up-regulation of HO-1 through MEK/ERK pathway. *Neuroscience Letters*.

[90] Garcia T. B., Hollborn M., Bringmann A. (2017). Expression and signaling of NGF in the healthy and injured retina. *Cytokine & Growth Factor Reviews*.

[91] Mysona B. A., Matragoon S., Stephens M. (2015). Imbalance of the nerve growth factor and its precursor as a potential biomarker for diabetic retinopathy. *BioMed Research International*.

[92] Simó R., Hernández C. (2014). Neurodegeneration in the diabetic eye: new insights and therapeutic perspectives. *Trends in Endocrinology & Metabolism*.

[93] Caillaud M., Chantemargue B., Richard L. (2018). Local low dose curcumin treatment improves functional recovery and remyelination in a rat model of sciatic nerve crush through inhibition of oxidative stress. *Neuropharmacology*.

[94] Eichler W., Savković-Cvijić H., Bürger S. (2017). Müller cell-derived PEDF mediates neuroprotection via STAT3 activation. *Cellular Physiology and Biochemistry*.

[95] Mishra B., Swaroop A., Kandpal R. P. (2016). Genetic components in diabetic retinopathy. *Indian Journal of Ophthalmology*.

[96] Zhang X., Saaddine J. B., Chou C. F. (2010). Prevalence of diabetic retinopathy in the United States, 2005-2008. *JAMA*.

[97] Simó-Servat O., Hernández C., Simó R. (2013). Genetics in diabetic retinopathy: current concepts and new insights. *Current Genomics*.

[98] Cho H., Sobrin L. (2014). Genetics of diabetic retinopathy. *Current Diabetes Reports*.

[99] Tom L., Davoudi S., Sobrin L. (2018). Genetic epidemiology of diabetic retinopathy. *Annals of Eye Science*.

[100] Owyong M., Schwartz S. G., Scott I. U. (2017). An update on the genetics of diabetic retinopathy. *Retina Today*.

[101] Pillai G. S., Varky R. (2016). Genetics in diabetic retinopathy - a brief review. *Kerala Journal of Ophthalmology*.

[102] Abhary S., Burdon K. P., Laurie K. J. (2010). Aldose reductase gene polymorphisms and diabetic retinopathy susceptibility. *Diabetes Care*.

[103] Ng D. P. K. (2010). Human genetics of diabetic retinopathy: current perspectives. *Journal of Ophthalmology*.

[104] Sun W., Oates P. J., Coutcher J. B., Gerhardinger C., Lorenzi M. (2006). A selective aldose reductase inhibitor of a new structural class prevents or reverses early retinal abnormalities in experimental diabetic retinopathy. *Diabetes*.

[105] Priščáková P., Minárik G., Repiská V. (2016). Candidate gene studies of diabetic retinopathy in human. *Molecular Biology Reports*.

[106] Tao D., Mai X., Zhang T., Mei Y. (2017). Association between the RAGE (receptor for advanced glycation end-products) -374T/A gene polymorphism and diabetic retinopathy in T2DM. *Revista da Associação Médica Brasileira*.

[107] Yu W., Yang J., Sui W., Qu B., Huang P., Chen Y. (2016). Association of genetic variants in the receptor for advanced glycation end products gene with diabetic retinopathy: a meta-analysis. *Medicine*.

[108] Buraczynska M., Ksiazek P., Baranowicz-Gaszczyk I., Jozwiak L. (2007). Association of the VEGF gene polymorphism with diabetic retinopathy in type 2 diabetes patients. *Nephrology Dialysis Transplantation*.

[109] Gonzalez-Salinas R., Garcia-Gutierrez M. C., Garcia-Aguirre G. (2017). Evaluation of VEGF gene polymorphisms and proliferative diabetic retinopathy in Mexican population. *International Journal of Ophthalmology*.

[110] Opatrilova R., Kubatka P., Caprnda M. (2018). Nitric oxide in the pathophysiology of retinopathy: evidences from preclinical and clinical researches. *Acta Ophthalmologica*.

[111] Yu C., Yi J., Yin X., Deng Y., Liao Y., Li X. (2015). Correlation of interactions between NOS3 polymorphisms and oxygen therapy with retinopathy of prematurity susceptibility. *International Journal of Clinical and Experimental Pathology*.

[112] Ma Z. J., Chen R., Ren H. Z., Guo X., Guo J., Chen L. M. (2014). Association between eNOS 4b/a polymorphism and the risk of diabetic retinopathy in type 2 diabetes mellitus: a meta-analysis. *Journal of Diabetes Research*.

[113] Momeni A., Chaleshtori M. H., Saadatmand S., Kheiri S. (2016). Correlation of endothelial nitric oxide synthase gene polymorphism (GG, TT and GT genotype) with proteinuria and retinopathy in type 2 diabetic patients. *Journal of Clinical and Diagnostic Research*.

[114] Zhao S., Li T., Zheng B., Zheng Z. (2012). Nitric oxide synthase 3 (NOS3) 4b/a, T-786C and G894T polymorphisms in association with diabetic retinopathy susceptibility: a meta-analysis. *Ophthalmic Genetics*.

[115] Wang Y., Ng M. C. Y., Lee S. C. (2003). Phenotypic heterogeneity and associations of two aldose reductase gene polymorphisms with nephropathy and retinopathy in type 2 diabetes. *Diabetes Care*.

[116] Santos K. G., Tschiedel B., Schneider J., Souto K., Roisenberg I. (2003). Diabetic retinopathy in Euro-Brazilian type 2 diabetic patients: relationship with polymorphisms in the aldose reductase, the plasminogen activator inhibitor-1 and the methylenetetrahydrofolate reductase genes. *Diabetes Research and Clinical Practice*.

[117] Cheema B. S., kohli H. S., Sharma R., Bhansali A., Khullar M. (2012). *Endothelial nitric oxide synthase* gene polymorphism and type 2 diabetic retinopathy among Asian Indians. *Acta Diabetologica*.

[118] Santos K. G., Crispim D., Canani L. H., Ferrugem P. T., Gross J. L., Roisenberg I. (2012). Relationship of endothelial nitric oxide synthase (*eNOS*) gene polymorphisms with diabetic retinopathy in Caucasians with type 2 diabetes. *Ophthalmic Genetics*.

[119] Ng Z. X., Kuppusamy U. R., Tajunisah I., Fong K. C. S., Chua K. H. (2012). Association analysis of −429T/C and −374T/A polymorphisms of receptor of advanced glycation end products (RAGE) gene in Malaysian with type 2 diabetic retinopathy. *Diabetes Research and Clinical Practice*.

[120] Vanita V. (2014). Association of RAGE (p.Gly82Ser) and MnSOD (p.Val16Ala) polymorphisms with diabetic retinopathy in T2DM patients from north India. *Diabetes Research and Clinical Practice*.

[121] Yang L., Wu Q., Li Y. (2013). Association of the receptor for advanced glycation end products gene polymorphisms and circulating RAGE levels with diabetic retinopathy in the Chinese population. *Journal of Diabetes Research*.

[122] Kangas-Kontio T., Vavuli S., Kakko S. J. (2009). Polymorphism of the manganese superoxide dismutase gene but not of vascular endothelial growth factor gene is a risk factor for diabetic retinopathy. *British Journal of Ophthalmology*.

[123] Abhary S., Burdon K. P., Gupta A. (2009). Common sequence variation in the *VEGFA* gene predicts risk of diabetic retinopathy. *Investigative Opthalmology & Visual Science*.

[124] Qiu M., Xiong W., Liao H., Li F. (2013). *VEGF* − 634G>C polymorphism and diabetic retinopathy risk: a meta-analysis. *Gene*.

[125] Dadbinpour A., Sheikhha M., Darbouy M., Afkhami-Ardekani M. (2013). Investigating *GSTT1* and *GSTM1* null genotype as the risk factor of diabetes type 2 retinopathy. *Journal of Diabetes & Metabolic Disorders*.

[126] Haghighi S. F., Salehi Z., Sabouri M. R., Abbasi N. (2015). Polymorphic variant of MnSOD A16V and risk of diabetic retinopathy. *Molecular Biology*.

[127] Fan W. Y., Liu N. P. (2015). Meta-analysis of association between K469E polymorphism of the ICAM-1 gene and retinopathy in type 2 diabetes. *International Journal of Ophthalmology*.

[128] Rodrigues K. F., Pietrani N. T., Sandrim V. C. (2015). Association of a large panel of cytokine gene polymorphisms with complications and comorbidities in type 2 diabetes patients. *Journal of Diabetes Research*.

[129] Bazzaz J. T., Amoli M. M., Taheri Z., Larijani B., Pravica V., Hutchinson I. V. (2014). TGF-*β*1 and IGF-I gene variations in type 1 diabetes microangiopathic complications. *Journal of Diabetes & Metabolic Disorders*.

[130] American Diabetes Association (2017). Introduction: standards of medical care in diabetes—2018. *Diabetes Care*.

[131] Martínez-González M. A., Salas-Salvadó J., Estruch R. (2015). Benefits of the Mediterranean diet: insights from the PREDIMED study. *Progress in Cardiovascular Diseases*.

[132] Díaz-López A., Babio N., Martínez-González M. A. (2015). Mediterranean diet, retinopathy, nephropathy, and microvascular diabetes complications: a post hoc analysis of a randomized trial. *Diabetes Care*.

[133] Chew E. Y. (2017). Dietary intake of omega-3 fatty acids from fish and risk of diabetic retinopathy. *JAMA*.

[134] PREDIMED study investigators, Tresserra-Rimbau A., Guasch-Ferré M. (2016). Intake of total polyphenols and some classes of polyphenols is inversely associated with diabetes in elderly people at high cardiovascular disease risk. *The Journal of Nutrition*.

[135] Guasch-Ferré M., Merino J., Sun Q., Fitó M., Salas-Salvadó J. (2017). Dietary polyphenols, Mediterranean diet, prediabetes, and type 2 diabetes: a narrative review of the evidence. *Oxidative Medicine and Cellular Longevity*.

[136] Pall M. L., Levine S. (2015). Nrf2, a master regulator of detoxification and also antioxidant, anti-inflammatory and other cytoprotective mechanisms, is raised by health promoting factors. *Sheng Li Xue Bao*.

[137] Boeing H., Bechthold A., Bub A. (2012). Critical review: vegetables and fruit in the prevention of chronic diseases. *European Journal of Nutrition*.

[138] Millen A. E., Sahli M. W., Nie J. (2016). Adequate vitamin D status is associated with the reduced odds of prevalent diabetic retinopathy in African Americans and Caucasians. *Cardiovascular Diabetology*.

[139] Beidokhti M. N., Jager A. K. (2017). Review of antidiabetic fruits, vegetables, beverages, oils and spices commonly consumed in the diet. *Journal of Ethnopharmacology*.

[140] Zafra-Stone S., Yasmin T., Bagchi M., Chatterjee A., Vinson J. A., Bagchi D. (2007). Berry anthocyanins as novel antioxidants in human health and disease prevention. *Molecular Nutrition & Food Research*.

[141] Millen A. E., Klein R., Folsom A. R., Stevens J., Palta M., Mares J. A. (2004). Relation between intake of vitamins C and E and risk of diabetic retinopathy in the Atherosclerosis Risk in Communities Study. *The American Journal of Clinical Nutrition*.

[142] Tanaka S., Yoshimura Y., Kawasaki R. (2013). Fruit intake and incident diabetic retinopathy with type 2 diabetes. *Epidemiology*.

[143] Sharma Y., Saxena S., Mishra A., Saxena A., Natu S. M. (2017). Nutrition for diabetic retinopathy: plummeting the inevitable threat of diabetic vision loss. *European Journal of Nutrition*.

[144] Kijlstra A., Tian Y., Kelly E. R., Berendschot T. T. J. M. (2012). Lutein: more than just a filter for blue light. *Progress in Retinal and Eye Research*.

[145] Ambati R., Phang S. M., Ravi S., Aswathanarayana R. (2014). Astaxanthin: sources, extraction, stability, biological activities and its commercial applications—a review. *Marine Drugs*.

[146] Neelam K., Goenadi C. J., Lun K., Yip C. C., Au Eong K. G. (2017). Putative protective role of lutein and zeaxanthin in diabetic retinopathy. *British Journal of Ophthalmology*.

[147] Gong X., Rubin L. P. (2015). Role of macular xanthophylls in prevention of common neovascular retinopathies: retinopathy of prematurity and diabetic retinopathy. *Archives of Biochemistry and Biophysics*.

[148] Su C. C., Chan C. M., Chen H. M. (2014). Lutein inhibits the migration of retinal pigment epithelial cells via cytosolic and mitochondrial Akt pathways (lutein inhibits RPE cells migration). *International Journal of Molecular Sciences*.

[149] Chao S. C., Vagaggini T., Nien C. W., Huang S. C., Lin H. Y. (2015). Effects of lutein and zeaxanthin on LPS-induced secretion of IL-8 by uveal melanocytes and relevant signal pathways. *Journal of Ophthalmology*.

[150] Silvan J. M., Reguero M., de Pascual-Teresa S. (2016). A protective effect of anthocyanins and xanthophylls on UVB-induced damage in retinal pigment epithelial cells. *Food & Function*.

[151] Murillo A. G., Fernandez M. L. (2016). Potential of dietary non-provitamin A carotenoids in the prevention and treatment of diabetic microvascular complications. *Advances in Nutrition*.

[152] Ishida S. (2009). Lifestyle-related diseases and anti-aging ophthalmology: suppression of retinal and choroidal pathologies by inhibiting renin-angiotensin system and inflammation. *Nippon Ganka Gakkai Zasshi*.

[153] Baccouche B., Benlarbi M., Barber A. J., Ben Chaouacha-Chekir R. (2018). Short-term administration of astaxanthin attenuates retinal changes in diet-induced diabetic *Psammomys obesus*. *Current Eye Research*.

[154] Dong L. Y., Jin J., Lu G., Kang X. L. (2013). Astaxanthin attenuates the apoptosis of retinal ganglion cells in *db/db* mice by inhibition of oxidative stress. *Marine Drugs*.

[155] Braakhuis A., Raman R., Vaghefi E. (2017). The association between dietary intake of antioxidants and ocular disease. *Diseases*.

[156] Kundu D., Mandal T., Nandi M., Osta M., Bandyopadhyay U., Ray D. (2014). Oxidative stress in diabetic patients with retinopathy. *Annals of African Medicine*.

[157] Young I. S., Torney J. J., Trimble E. R. (1992). The effect of ascorbate supplementation on oxidative stress in the streptozotocin diabetic rat. *Free Radical Biology and Medicine*.

[158] Gupta M. M., Chari S. (2005). Lipid peroxidation and antioxidant status in patients with diabetic retinopathy. *Indian Journal of Physiology and Pharmacology*.

[159] Vinson J., Hsu C., Possanza C. (1994). Lipid peroxidation and diabetic complications: effect of antioxidant vitamins C and E. *Free Radicals in Diagnostic Medicine*.

[160] Ulker E., Parker W. H., Raj A., Qu Z. C., May J. M. (2016). Ascorbic acid prevents VEGF-induced increases in endothelial barrier permeability. *Molecular and Cellular Biochemistry*.

[161] Aruoma O. I., Halliwell B. (1987). Superoxide-dependent and ascorbate-dependent formation of hydroxyl radicals from hydrogen peroxide in the presence of iron. Are lactoferrin and transferrin promoters of hydroxyl-radical generation?. *Biochemical Journal*.

[162] Wang H., Zhang Z. B., Wen R. R., Chen J. W. (1995). Experimental and clinical studies on the reduction of erythrocyte sorbitol-glucose ratios by ascorbic acid in diabetes mellitus. *Diabetes Research and Clinical Practice*.

[163] May J. M., Jayagopal A., Qu Z. C., Parker W. H. (2014). Ascorbic acid prevents high glucose-induced apoptosis in human brain pericytes. *Biochemical and Biophysical Research Communications*.

[164] Fukui M., Yamabe N., Choi H. J., Polireddy K., Chen Q., Zhu B. T. (2015). Mechanism of ascorbate-induced cell death in human pancreatic cancer cells: role of Bcl-2, beclin 1 and autophagy. *Planta Medica*.

[165] Martin A., Joseph J. A., Cuervo A. M. (2002). Stimulatory effect of vitamin C on autophagy in glial cells. *Journal of Neurochemistry*.

[166] Hung T. H., Chen S. F., Li M. J., Yeh Y. L., Hsieh T.'. T.'. (2010). Differential effects of concomitant use of vitamins C and E on trophoblast apoptosis and autophagy between normoxia and hypoxia-reoxygenation. *PLoS One*.

[167] Wang X., Quinn P. J. (1999). Vitamin E and its function in membranes. *Progress in Lipid Research*.

[168] Jain S. K., Palmer M. (1997). The effect of oxygen radicals metabolites and vitamin E on glycosylation of proteins. *Free Radical Biology and Medicine*.

[169] Chung T. W., Hau Yu J. J., Liu D. Z. (1998). Reducing lipid peroxidation stress of erythrocyte membrane by *α*-tocopherol nicotinate plays an important role in improving blood rheological properties in type 2 diabetic patients with retinopathy. *Diabetic Medicine*.

[170] Schleicher E. D., Wagner E., Nerlich A. G. (1997). Increased accumulation of the glycoxidation product N(epsilon)-(carboxymethyl)lysine in human tissues in diabetes and aging. *Journal of Clinical Investigation*.

[171] De Pascale M. C., Bassi A. M., Patrone V., Villacorta L., Azzi A., Zingg J.-M. (2006). Increased expression of transglutaminase-1 and PPAR*γ* after vitamin E treatment in human keratinocytes. *Archives of Biochemistry and Biophysics*.

[172] Azzi A., Boscoboinik D., Clément S. (1997). *α*-tocopherol as a modulator of smooth muscle cell proliferation. *Prostaglandins, Leukotrienes and Essential Fatty Acids*.

[173] Kunisaki M., Bursell S. E., Clermont A. C. (1995). Vitamin E prevents diabetes-induced abnormal retinal blood flow via the diacylglycerol-protein kinase C pathway. *American Journal of Physiology-Endocrinology and Metabolism*.

[174] Nakagawa K., Shibata A., Yamashita S. (2007). In vivo angiogenesis is suppressed by unsaturated vitamin E, tocotrienol. *The Journal of Nutrition*.

[175] Wong M. Y. Z., Man R. E. K., Fenwick E. K. (2018). Dietary intake and diabetic retinopathy: a systematic review. *PLoS One*.

[176] Rodríguez-Carrizalez A. D., Castellanos-González J. A., Martínez-Romero E. C. (2016). The effect of ubiquinone and combined antioxidant therapy on oxidative stress markers in non-proliferative diabetic retinopathy: a phase IIa, randomized, double-blind, and placebo-controlled study. *Redox Report*.

[177] Jarosz M., Olbert M., Wyszogrodzka G., Młyniec K., Librowski T. (2017). Antioxidant and anti-inflammatory effects of zinc. Zinc-dependent NF-*κ*B signaling. *Inflammopharmacology*.

[178] Kogan S., Sood A., Garnick M. S. (2017). Zinc and wound healing: a review of zinc physiology and clinical applications. *Wounds*.

[179] Balsano C., Porcu C., Sideri S. (2018). Is copper a new target to counteract the progression of chronic diseases?. *Metallomics*.

[180] de Figueiredo Ribeiro S. M., Braga C. B. M., Peria F. M. (2016). Effect of zinc supplementation on antioxidant defenses and oxidative stress markers in patients undergoing chemotherapy for colorectal cancer: a placebo-controlled, prospective randomized trial. *Biological Trace Element Research*.

[181] Guo C. H., Wang C. L. (2013). Effects of zinc supplementation on plasma copper/zinc ratios, oxidative stress, and immunological status in hemodialysis patients. *International Journal of Medical Sciences*.

[182] Ranasinghe P., Pigera S., Galappatthy P., Katulanda P., Constantine G. R. (2015). Zinc and diabetes mellitus: understanding molecular mechanisms and clinical implications. *DARU Journal of Pharmaceutical Sciences*.

[183] Barman S., Srinivasan K. (2017). Attenuation of oxidative stress and cardioprotective effects of zinc supplementation in experimental diabetic rats. *British Journal of Nutrition*.

[184] Fujimoto S., Yasui H., Yoshikawa Y. (2013). Development of a novel antidiabetic zinc complex with an organoselenium ligand at the lowest dosage in KK-A^y^ mice. *Journal of Inorganic Biochemistry*.

[185] Sharif R., Thomas P., Zalewski P., Fenech M. (2015). Zinc supplementation influences genomic stability biomarkers, antioxidant activity, and zinc transporter genes in an elderly Australian population with low zinc status. *Molecular Nutrition & Food Research*.

[186] Cousins R. J., Blanchard R. K., Moore J. B. (2003). Regulation of zinc metabolism and genomic outcomes. *The Journal of Nutrition*.

[187] Bray T. M., Bettger W. J. (1990). The physiological role of zinc as an antioxidant. *Free Radical Biology and Medicine*.

[188] Lee S. R. (2018). Critical role of zinc as either an antioxidant or a prooxidant in cellular systems. *Oxidative Medicine and Cellular Longevity*.

[189] Afshar Ebrahimi F., Foroozanfard F., Aghadavod E., Bahmani F., Asemi Z. (2018). The effects of magnesium and zinc co-supplementation on biomarkers of inflammation and oxidative stress, and gene expression related to inflammation in polycystic ovary syndrome: a randomized controlled clinical trial. *Biological Trace Element Research*.

[190] Liu Z., Wu X., Zhang T. (2015). Effects of dietary copper and zinc supplementation on growth performance, tissue mineral retention, antioxidant status, and fur quality in growing-furring blue foxes (*Alopex lagopus*). *Biological Trace Element Research*.

[191] Gomes M. B., Negrato C. A. (2014). Alpha-lipoic acid as a pleiotropic compound with potential therapeutic use in diabetes and other chronic diseases. *Diabetology & Metabolic Syndrome*.

[192] Rochette L., Ghibu S., Muresan A., Vergely C. (2015). Alpha-lipoic acid: molecular mechanisms and therapeutic potential in diabetes. *Canadian Journal of Physiology and Pharmacology*.

[193] Packer L. (1994). Antioxidant properties of lipoic acid and its therapeutic effects in prevention of diabetes complications and cataractsa. *Annals of the New York Academy of Sciences*.

[194] Suh J. H., Shenvi S. V., Dixon B. M. (2004). Decline in transcriptional activity of Nrf2 causes age-related loss of glutathione synthesis, which is reversible with lipoic acid. *Proceedings of the National Academy of Sciences of the United States of America*.

[195] Biewenga G. P., Haenen G. R. M. M., Bast A. (1997). The pharmacology of the antioxidant lipoic acid. *General Pharmacology: The Vascular System*.

[196] Lee S. G., Lee C. G., Yun I. H., Hur D. Y., Yang J. W., Kim H. W. (2012). Effect of lipoic acid on expression of angiogenic factors in diabetic rat retina. *Clinical & Experimental Ophthalmology*.

[197] Kan E., Alici Ö., Kan E. K., Ayar A. (2017). Effects of alpha-lipoic acid on retinal ganglion cells, retinal thicknesses, and VEGF production in an experimental model of diabetes. *International Ophthalmology*.

[198] Artwohl M., Muth K., Kosulin K. (2007). R-(+)-*α*-lipoic acid inhibits endothelial cell apoptosis and proliferation: involvement of Akt and retinoblastoma protein/E2F-1. *American Journal of Physiology-Endocrinology and Metabolism*.

[199] Chen C.-L., Cheng W. S., Chen J. L., Chiang C. H. (2013). Potential of nonoral *α*-lipoic acid aqueous formulations to reduce ocular microvascular complications in a streptozotocin-induced diabetic rat model. *Journal of Ocular Pharmacology and Therapeutics*.

[200] Santos J. M., Kowluru R. A. (2011). Role of mitochondria biogenesis in the metabolic memory associated with the continued progression of diabetic retinopathy and its regulation by lipoic acid. *Investigative Opthalmology & Visual Science*.

[201] Nebbioso M., Pranno F., Pescosolido N. (2013). Lipoic acid in animal models and clinical use in diabetic retinopathy. *Expert Opinion on Pharmacotherapy*.

[202] Lin J., Bierhaus A., Bugert P. (2006). Effect of R-(+)-*α*-lipoic acid on experimental diabetic retinopathy. *Diabetologia*.

[203] Nebbioso M., Federici M., Rusciano D., Evangelista M., Pescosolido N. (2012). Oxidative stress in preretinopathic diabetes subjects and antioxidants. *Diabetes Technology & Therapeutics*.

[204] Haritoglou C., Gerss J., Hammes H. P., Kampik A., Ulbig M. W. (2011). Alpha-lipoic acid for the prevention of diabetic macular edema. *Ophthalmologica*.

[205] Peres T. V., Schettinger M. R. C., Chen P. (2016). Manganese-induced neurotoxicity: a review of its behavioral consequences and neuroprotective strategies. *BMC Pharmacology and Toxicology*.

[206] Sigel A., Sigel H., Sigel R. K. O. (2013). *Interrelations between essential metal ions and human diseases*.

[207] Salmonowicz B., Krzystek-Korpacka M., Noczyńska A. (2014). Trace elements, magnesium, and the efficacy of antioxidant systems in children with type 1 diabetes mellitus and in their siblings. *Advances in Clinical and Experimental Medicine*.

[208] Martinez-Finley E. J., Gavin C. E., Aschner M., Gunter T. E. (2013). Manganese neurotoxicity and the role of reactive oxygen species. *Free Radical Biology and Medicine*.

[209] Aguirre J. D., Culotta V. C. (2012). Battles with iron: manganese in oxidative stress protection. *Journal of Biological Chemistry*.

[210] Lee S. H., Jouihan H. A., Cooksey R. C. (2013). Manganese supplementation protects against diet-induced diabetes in wild type mice by enhancing insulin secretion. *Endocrinology*.

[211] Lestari M. L. A. D., Indrayanto G. (2014). Chapter Three - Curcumin. *Profiles of Drug Substances, Excipients and Related Methodology*.

[212] Joint FAO/WHO Expert Committee on Food Additives (2004). Evaluation of certain food additives and contaminants. *World Health Organ Tech Rep Ser*.

[213] Priyadarsini K. I., Maity D. K., Naik G. H. (2003). Role of phenolic O-H and methylene hydrogen on the free radical reactions and antioxidant activity of curcumin. *Free Radical Biology and Medicine*.

[214] Pinlaor S., Yongvanit P., Prakobwong S. (2009). Curcumin reduces oxidative and nitrative DNA damage through balancing of oxidant-antioxidant status in hamsters infected with *Opisthorchis viverrini*. *Molecular Nutrition & Food Research*.

[215] Molina-Jijón E., Tapia E., Zazueta C. (2011). Curcumin prevents Cr(VI)-induced renal oxidant damage by a mitochondrial pathway. *Free Radical Biology and Medicine*.

[216] Kowluru R. A., Kanwar M. (2007). Effects of curcumin on retinal oxidative stress and inflammation in diabetes. *Nutrition & Metabolism*.

[217] Gupta S. K., Kumar B., Nag T. C. (2011). Curcumin prevents experimental diabetic retinopathy in rats through its hypoglycemic, antioxidant, and anti-inflammatory mechanisms. *Journal of Ocular Pharmacology and Therapeutics*.

[218] Flynn D. L., Rafferty M. F., Boctor A. M. (1986). Inhibition of 5-hydroxy-eicosatetraenoic acid (5-HETE) formation in intact human neutrophils by naturally-occurring diarylheptanoids: inhibitory activities of curcuminoids and yakuchinones. *Prostaglandins, Leukotrienes and Medicine*.

[219] Sameermahmood Z., Balasubramanyam M., Saravanan T., Rema M. (2008). Curcumin modulates SDF-1*α*/CXCR4–induced migration of human retinal endothelial cells (HRECs). *Investigative Opthalmology & Visual Science*.

[220] Mandal M. N. A., Patlolla J. M. R., Zheng L. (2009). Curcumin protects retinal cells from light-and oxidant stress-induced cell death. *Free Radical Biology and Medicine*.

[221] Li Y., Zou X., Cao K. (2013). Curcumin analog 1, 5-bis (2-trifluoromethylphenyl)-1, 4-pentadien-3-one exhibits enhanced ability on Nrf2 activation and protection against acrolein-induced ARPE-19 cell toxicity. *Toxicology and Applied Pharmacology*.

[222] Nabavi S. F., Thiagarajan R., Rastrelli L. (2015). Curcumin: a natural product for diabetes and its complications. *Current Topics in Medicinal Chemistry*.

[223] Wang L. L., Sun Y., Huang K., Zheng L. (2013). Curcumin, a potential therapeutic candidate for retinal diseases. *Molecular Nutrition & Food Research*.

[224] Yoshino M., Haneda M., Naruse M. (2004). Prooxidant activity of curcumin: copper-dependent formation of 8-hydroxy-2′-deoxyguanosine in DNA and induction of apoptotic cell death. *Toxicology in Vitro*.

[225] Wallace T. C., Giusti M. M. (2015). Anthocyanins. *Advances in Nutrition*.

[226] Lila M. A., Burton-Freeman B., Grace M., Kalt W. (2016). Unraveling anthocyanin bioavailability for human health. *Annual Review of Food Science and Technology*.

[227] Fang J. (2014). Bioavailability of anthocyanins. *Drug Metabolism Reviews*.

[228] Skrovankova S., Sumczynski D., Mlcek J., Jurikova T., Sochor J. (2015). Bioactive compounds and antioxidant activity in different types of berries. *International Journal of Molecular Sciences*.

[229] Wang H., Nair M. G., Strasburg G. M. (1999). Antioxidant and antiinflammatory activities of anthocyanins and their aglycon, cyanidin, from tart cherries. *Journal of Natural Products*.

[230] Aboonabi A., Singh I. (2015). Chemopreventive role of anthocyanins in atherosclerosis via activation of Nrf2–ARE as an indicator and modulator of redox. *Biomedicine & Pharmacotherapy*.

[231] Vendrame S., Klimis-Zacas D. (2015). Anti-inflammatory effect of anthocyanins via modulation of nuclear factor-*κ*B and mitogen-activated protein kinase signaling cascades. *Nutrition Reviews*.

[232] Mena P., Domínguez-Perles R., Gironés-Vilaplana A., Baenas N., García-Viguera C., Villaño D. (2014). Flavan-3-ols, anthocyanins, and inflammation. *IUBMB Life*.

[233] Guo H., Ling W. (2015). The update of anthocyanins on obesity and type 2 diabetes: experimental evidence and clinical perspectives. *Reviews in Endocrine and Metabolic Disorders*.

[234] Žmitek K., Pogačnik T., Mervic L., Žmitek J., Pravst I. (2017). The effect of dietary intake of coenzyme Q10 on skin parameters and condition: results of a randomised, placebo-controlled, double-blind study. *Biofactors*.

[235] Lim S. C., Tan H. H., Goh S. K. (2006). Oxidative burden in prediabetic and diabetic individuals: evidence from plasma coenzyme Q_10_. *Diabetic Medicine*.

[236] Garrido-Maraver J., Cordero M. D., Oropesa-Avila M. (2014). Clinical applications of coenzyme Q_10_. *Frontiers in Bioscience*.

[237] Modi K., Santani D. D., Goyal R. K., Bhatt P. A. (2006). Effect of coenzyme Q10 on catalase activity and other antioxidant parameters in streptozotocin-induced diabetic rats. *Biological Trace Element Research*.

[238] Dhanasekaran M., Ren J. (2005). The emerging role of coenzyme Q-10 in aging, neurodegeneration, cardiovascular disease, cancer and diabetes mellitus. *Current Neurovascular Research*.

[239] Li J., Yu S., Ying J., Shi T., Wang P. (2017). Resveratrol prevents ROS-induced apoptosis in high glucose-treated retinal capillary endothelial cells via the activation of AMPK/Sirt1/PGC-1*α* pathway. *Oxidative Medicine and Cellular Longevity*.

[240] Abu-Amero K. K., Kondkar A. A., Chalam K. V. (2016). Resveratrol and ophthalmic diseases. *Nutrients*.

[241] Goldberg D. M., Yan J., Soleas G. J. (2003). Absorption of three wine-related polyphenols in three different matrices by healthy subjects. *Clinical Biochemistry*.

[242] Walle T. (2011). Bioavailability of resveratrol. *Annals of the New York Academy of Sciences*.

[243] Doganay S., Borazan M., Iraz M., Cigremis Y. (2006). The effect of resveratrol in experimental cataract model formed by sodium selenite. *Current Eye Research*.

[244] Hightower K. R., McCready J. P. (1991). Effect of selenite on epithelium of cultured rabbit lens. *Investigative Ophthalmology & Visual Science*.

[245] Bola C., Bartlett H., Eperjesi F. (2014). Resveratrol and the eye: activity and molecular mechanisms. *Graefe's Archive for Clinical and Experimental Ophthalmology*.

[246] Kim Y. H., Kim Y. S., Roh G. S., Choi W. S., Cho G. J. (2012). Resveratrol blocks diabetes-induced early vascular lesions and vascular endothelial growth factor induction in mouse retinas. *Acta Ophthalmologica*.

[247] Luna C., Li G., Liton P. B. (2009). Resveratrol prevents the expression of glaucoma markers induced by chronic oxidative stress in trabecular meshwork cells. *Food and Chemical Toxicology*.

[248] Gong Y., Fu Z., Liegl R., Chen J., Hellström A., Smith L. E. H. (2017). *ω*-3 and *ω*-6 long-chain PUFAs and their enzymatic metabolites in neovascular eye diseases. *The American Journal of Clinical Nutrition*.

[249] Behl T., Kotwani A. (2017). Omega-3 fatty acids in prevention of diabetic retinopathy. *Journal of Pharmacy and Pharmacology*.

[250] Dátilo M. N., Sant’Ana M. R., Formigari G. P. (2018). Omega-3 from flaxseed oil protects obese mice against diabetic retinopathy through GPR120 receptor. *Scientific Reports*.

[251] Sala-Vila A., Díaz-López A., Valls-Pedret C. (2016). Dietary marine *ω*-3 fatty acids and incident sight-threatening retinopathy in middle-aged and older individuals with type 2 diabetes: prospective investigation from the PREDIMED trial. *JAMA Ophthalmology*.

[252] Sapieha P., Chen J., Stahl A. (2012). Omega-3 polyunsaturated fatty acids preserve retinal function in type 2 diabetic mice. *Nutrition & Diabetes*.

